# Lipin3 deficiency aggravates cisplatin induced acute kidney injury via activating Sirt1-p21-Caspase 3-GSDME pyroptosis pathway

**DOI:** 10.7150/ijbs.110125

**Published:** 2025-08-11

**Authors:** Yu-Xing Liu, Hao Huang, Fang Wang, Mei-Fang Zhao, Jie-Yuan Jin, Yi Dong, Qian Wang, Liang-Liang Fan, Rong Xiang

**Affiliations:** 1 Department of Nephrology, Xiangya Hospital, Central South University, Changsha, 410013, China.; 2 Department of cell biology, School of Life Sciences, Central South University, Changsha, 410013, China.; 3 Hunan Key Laboratory of Animal Models for Human Diseases, School of Life Sciences, Central South University, Changsha, 410013, China.; 4 Department of Endocrinology, The Third Xiangya Hospital of Central South University, Changsha, Hunan 410013, China.

**Keywords:** acute kidney injury, Lipin3, pyroptosis, GSDME

## Abstract

Lipin proteins, including Lipin 1, Lipin 2 and Lipin3, play a vital role in lipid metabolism. Despite their significance, there is limited understanding of the involvement of Lipin proteins in kidney diseases. This study aims to elucidate the specific functions of Lipin 3 in the context of acute kidney injury (AKI). In the present study, Lipin3 levels were analyzed in AKI public database, kidney tissues from AKI patients and cisplatin induced mice models, as well as cisplatin induced HK2 cells. A *Lipin3* knockout (*Lipin3*-KO) mouse model was generated to investigate the pathophysiological roles of Lipin3 in the kidneys. The underlying mechanisms were further examined in primary tubular epithelial cells (PTECs) and HK2 cells *in vitro*. The findings indicated that (1) Lipin3 was obviously increased in AKI patients, as well as cisplatin induced mice and cells; (2) Lipin3-null mice presented with more severe AKI symptoms compared to WT mice after cisplatin treatment; (3) Lipin3 played crucial role in regulating cell death and mitochondrial function after cisplatin treatment; (4) In terms of mechanism, Lipin3 regulated these phenotypes through its interaction with Sirt1, which activated the p21-Caspase 3-GSDME pathway. Our study suggests that Lipin3 could be pivotal in pyroptosis and AKI. Decreased Lipin3 levels in the kidney may potentially contribute as a risk factor for exacerbating AKI.

## Introduction

Acute kidney injury (AKI) is characterized by a sudden decrease in glomerular filtration rate, manifested by a remarkable increase in serum creatinine levels (≥0.3 mg/dl within 2 days) and a reduction in urinary output.^1^-^3^ Recent clinical guidelines from Kidney Disease Improving Global Outcomes revealed that AKI has emerged as a significant global public health concern, impacting millions of patients and resulting in reduced survival rates and heightened progression of underlying chronic kidney disease.^4^-^6^ Broadly, AKI incidence is categorized as either community-acquired or hospital-acquired. Hospital-acquired AKI is primarily attributed to post-surgical or diagnostic interventions, as well as iatrogenic factors. On the other hand, community-acquired AKI is associated with significant factors such as sepsis, volume depletion, toxins, and pregnancy.^7^,^8^ The sepsis-induced AKI model and cisplatin induced AKI model are the main types of animal models in AKI studies.^9^,^10^ Preclinical investigations have revealed cellular and molecular mechanisms underlying cisplatin-induced AKI. These mechanisms encompass intracellular stresses such as DNA damage, mitochondrial pathology, oxidative stress and endoplasmic reticulum (ER) stress.^11^

The Lipin protein family is a class of dual-function metabolic regulators that act both as phosphatidate phosphatases (PAP) involved in lipid biosynthesis and as coactivators of gene expression in the nucleus. This family is highly conserved among eukaryotes, and its members play a central role in the Kennedy pathway of glycerophospholipid metabolism by catalyzing the dephosphorylation of phosphatidate (PA) to diacylglycerol (DAG). In addition, Lipins can translocate to the nucleus and work in concert with transcription factors to regulate the expression of genes involved in mitochondrial oxidative metabolism and fatty acid β-oxidation.^12^ The Lipin protein family, comprising Lipin1, Lipin2, and Lipin3 in mammalian systems, possesses two highly conserved domains: the amino-terminal Lipin (N-LIP) domain and the carboxy-terminal Lipin (C-LIP) domain. The N-LIP domain is crucial for catalytic activity, nuclear localization, and binding to protein phosphatase-1cγ. Meanwhile, the C-LIP domain regulates both PAP enzyme activity and transcriptional co-activator function.^13^ Prior researches have underscored the Lipin protein family's significant roles in lipid metabolism, inflammatory responses and cell differentiation.^14^,^15^ While Lipin1 has been extensively studied, the functions of Lipin2 and Lipin3 remain less elucidated in current research. Simultaneously, the function of Lipin protein family in kidney were also not clear.

In this study, we observed a substantial increase in Lipin3 expression in AKI patients, cisplatin-treated mice and HK-2 cells, as well as human-derived renal organoids. The Lipin3-null mice exhibited severe symptoms including higher levels of creatinine, urea nitrogen and tubular damage scores compared to wild type (WT) mice after cisplatin treatment. Additional functional studies showed that, as a transcriptional co-activator, Lipin3 can interact with Sirt1 in cell nucleus. After cisplatin treatment, the Lipin3 deficiency can reduce the ability of Sirt1 in repressing p21, which leads to the overexpression of p21 and further activates the Caspase 3 and GSDME, finally resulting in mitochondrial dysfunction, apoptosis and Caspase 3/GSDME‑dependent secondary pyroptosis in primary tubular epithelial cells (PTECs) and aggravated cisplatin induced AKI. Therefore, our study suggests that Lipin3 might play a vital role in AKI, and a reduction in Lipin3 within the kidney could potentially be a risk factor for AKI.

## Materials and Methods

Research involving human subjects complied with all relevant national regulations, institutional policies and is in accordance with the tenets of the Helsinki Declaration (as revised in 2013), and has been approved by the authors' Institutional Review Board (the Third XiangYa hospital of Central Soth University, approval No. 2020-S533, date 2020.9.15). Informed consent was obtained from all individuals included in this study.

### Key reagents, Mouse strains, cell lines and human tissue samples

The cisplatin (D8810) was procured from Beijing Solarbio Science & Technology Co., Ltd. JNK inhibitors, SP600125 (S1460) was purchased from Selleck. The Lipin3 antibody was generated by Shanghai YouKe Biotechnology Co., Ltd. Antibodies against GSDMD (20770-1-AP) and Fis1 (10956-1-AP) were purchased from Proteintech Group, Inc. Antibody against MFN2 (abs137583a) and OPA1 (abs138107) was procured from Absin bioscience Inc. Antibody against p21 (R25235) were procured from Zhengneng Biotechnology Co., Ltd (ZEN-BIO). Antibodies against NLRP3 (# D4D8T) GSDME (# 19453S), Caspase-3 (# 9665S), β-actin (# 4970S) and Sirt1(# 2493S) were procured from Cell Signaling Technology. Antibodies against Caspase 1 (EPR16883) was procured from Abcam. OxPhos rodent WB antibody cocktail (# 45-8099) was procured from Invitrogen. A BCA Protein Assay and Analysis Kit (23227), a PureLink® RNA Mini Kit (12183025), a RevertAid First Strand cDNA Synthesis Kit (K1621) and a Maxima SYBR Green/ROX qPCR Master Mix (2×) (K0221) were purchased from Thermo Fisher Scientific. A Urea Nitrogen (BUN) Colorimetric Detection Kit (EIABUN) was purchased from Novo Biotechnology Co., Ltd. A Creatinine Colorimetric Detection Kit (BC4910), a Hematoxylin-Eosin/HE Staining Kit (G1120), a Cell counting Kit-8 (CCK8 CK04), a Reactive Oxygen Species (ROS) Assay Kit (CA1410), an Adenosine triphosphate (ATP) Assay Kit (BC0300), a Mitochondrial Membrane Potential Assay Kit with JC-1 (M8650) and a Broad Spectrum Immunohistochemistry Kit (SP0041) were procured from Beijing Solarbio Science & Technology Co., Ltd. The Lactate dehydrogenase (LDH) activity assay kit (AKCO003M) was purchased from Beijing Boxbio Science & Technology Co., Ltd. Mouse IL-6 ELISA Kit (JL20268) and mouse IL-18 ELISA Kit (JL20253) were obtained from Jianglai Biology. Human Lipin3 ELISA Kit (F9970-A) was obtained from Kexing Biology.

*In vivo Lipin3* gene overexpression was achieved by AAV9 vectors 16-18. Recombinant AAV9 vectors carrying Lipin3 or Empty vector with a pCMV promoter (AAV-Lipin3 or AAV9-Empty) were manufactured by GeneChem Co, Ltd (Shanghai, China). AAV9-Empty served as negative control. AAV9-Lipin3/Empty vectors (1 × 10^12^ copies per mouse) were delivered by tail vein injection. Four weeks after AAV delivery, Lipin3 overexpression was verified by Western blot.

*Lipin3* knockout (*Lipin3*-KO) mice with exon 3-7 deletion were generated by Cyagen (Suzhou, China), and genotyping was conducted following established procedures.^19^ C57BL/6J mice were obtained from the Chinese Academy of Science (Shanghai, China) and bred in the Department of Zoology, Central South University. To establish cisplatin induced-AKI mice model, *Lipin3*-KO mice and WT control littermates aged 8 weeks were intraperitoneal injection with 20 mg/kg cisplatin one time. The control mice were administered the same volume of 0.9% saline. After 3 days cisplatin administration, mice were sacrificed on the 4th day. Blood and kidney samples were collected for subsequent analysis.

For the intervention experiment, *Lipin3*-KO mice were intraperitoneal injection with SP600125 (10 mg/kg, in 5% DMSO + 30% PEG300 + 5% Tween 80) for 1 h before cisplatin administration. The control mice were administered the same volume of 5% DMSO + 30% PEG300 + 5%Tween 80 or 0.9% saline. After 3 days cisplatin administration, mice were sacrificed on the 4th day.

To observe the effect of *Lipin3* on the survival rate of cisplatin‑induced AKI mice, 40 *Lipin3*-KO mice and 40 WT control mice were intraperitoneally injected with 20 mg/kg cisplatin one time. The survival was observed every day until the 10th day and survival curves were then plotted.

PTECs were isolated from mouse kidneys following a previously described protocol.^20^ Briefly, the procedure involved: 1) decapsulation and bisecting of kidneys, removing the medulla; 2) finely chopping the remaining cortices and digesting them in 1 mg/ml collagenase type-II at 37°C for 10 minutes; 3) passing the kidney digest through a series of brass sieves with decreasing mesh openings; 4) collecting cells from a 40 µm nylon mesh and centrifuging at 800 rpm for 10 minutes; and 5) resuspending the cell pellet in a medium selective for epithelial cell growth, seeding it onto 1% gelatin-coated tissue culture plates, and incubating at 37°C with 5% CO_2_.

The human proximal tubular epithelial cell line, HK2 cells were acquired from the Cell Bank of Shanghai Institutes for Biological Sciences (Shanghai, China), was cultured at 37°C in a humidified, 5% CO_2_-controlled atmosphere. The culture medium used was DMEM supplemented with 10% fetal bovine serum, 50 IU/mL penicillin, 50 mmol/L streptomycin, and glutamine. The cells were stimulated with cisplatin (20 μg/ml) to induce cell death, with or without pretreatment with SP600125 (20 μM) for 1 h.

The study protocol received approval from the Review Board of Central South University in China. The studies involving human participants and animals were reviewed and approved by The Third Xiangya Hospital of the Central South University Ethics Committee (approval No. 2020-S533, date 2020.9.15). Kidney tissues were obtained from puncture or surgical specimen, healthy kidney tissues were enrolled from patients with renal contusion. All patients provided written informed consent.

### Data source

The data were acquired from the Gene Expression Omnibus (GEO) database (http://www.ncbi.nlm.nih.gov/geo/). The search words “AKI” or “cisplatin” and “human” and “Expression profiling by array” were applied for dataset retrieval. After screening, GSE106993,^21^ GSE145085^22^ and GSE164647 were selected for subsequent bioinformatics analysis.

### Bioinformatics analysis

For differentially expressed genes (DEGs) analysis, genes with an expression adjusted-*p* value < 0.05 and log2 (|fold change|) > 1 were regarded as significant DEGs. GO Enrichment and KEGG analysis of DEGs were performed using the “clusterProfiler” (version 3.18.1) R package, as described previously.^23^ Signal pathways with a *p*-value less than 0.05 and an enrichment score greater than 0.75 are considered significant signal pathways. Single-cell data analysis using the R package “Seurat” (version 4.0.2), as described previously.^24^,^25^ Gene set enrichment analysis (GSEA) was performed as described previously. 23,24

### Biochemical analysis

Serum was separated from whole blood after centrifugation. Activities of BUN and creatinine in serum were respectively detected by using assay kits according to the manufacturer's instruction.

### HE staining and Immunohistochemistry

Paraformaldehyde-fixed kidney tissue was embedded in paraffin and sliced into 3 μm sections. The sections were stained with a HE staining kit and examined by routine light microscopy. Staining was performed according to established protocols. The renal injury scores were assigned in accordance with the methodology from prior research.^26^,^27^

For immunohistochemistry experiments on human kidney tissues, an immunohistochemistry kit was used. The tissues were sectioned in the sagittal plane at a thickness of 5 μm using a cryostat after fixation in 4% paraformaldehyde and embedding in optimal cutting temperature compound.

### Terminal deoxynucleotidyl transferase dUTP Nick end labeling (TUNEL)

Cell apoptosis was assessed using the TUNEL assay kit (Promega, G3250) following the manufacturer's instructions. Apoptotic tissue, indicated by localized green fluorescence, was observed using a fluorescent microscope (Leica, DM4000B).

### Western Blot (WB) Analysis and Co-immunoprecipitation (co-IP)

For WB analysis, the cortical part of the kidney tissues or cells were homogenized on ice in RIPA lysis buffer (P0013K, Beyotime) containing complete protease inhibitors (Roche Bioscience, No. 04693159001) and 0.1 mmol/L Na_3_VO_4_ to inhibit phosphatase. After centrifugation at 15 000 g for 10 minutes, the supernatants were collected, and the protein concentration was measured with the BCA kit following the manufacturer's instructions. Equal amounts of protein lysates were resolved on 8% to 12% Bis-Tris NuPAGE gels, followed by standard Western blotting with the antibodies specified above. Chemiluminescent signals were scanned, and integrated density values were calculated with a chemiluminescent imaging system (Alpha Innotech).

For co-IP, the kidney tissues of WT mice were lysed, and equal amounts of lysates (500 μg in 1 mL) were used for immunoprecipitation with Protein A+G beads (P2108, Beyotime) overnight. The extensively washed immunoprecipitates were resolved on an 8% to 12% NuPage Bis-Tris gel, followed by standard Western blotting with the antibodies specified above.

### Immunofluorescence confocal microscopy

For confocal imaging, the cultured cells were fixed with 4% paraformaldehyde and treated with 0.5% Triton X-100. Staining with specific antibodies was performed, and the samples were examined on the Leica SP5 platform following standard procedures.

### Transfection

The wild-type (WT) Sirt1 CDS with C-terminal Flag-tag in the pEnter was designed. The sense small interfering RNA (siRNA) sequence targeting p21 was as follows: 5'-GAUGGAACUUCGACUUUGUTT-3' (GenePharma, Shanghai, China). The cells were transiently transfected with Sirt1-Flag-pEnter or Flag-pEnter or scrambled siRNA or *p21* siRNA using Lipofectamine™ 2000 CD Transfection Reagent (Thermo Fisher Scientific) following the manufacturer's instructions.

### ATP assay, ROS assay and JC-1 staining

ATP assays and ROS assays were performed using respective assay kits. Phosphomolybdic acid colorimetry was employed to detect the levels of ATP. The generation of ROS was determined by fluorometric analysis using 2,7-dichlorofluorescin diacetate (DCFH-DA). For Mitochondrial membrane potential, the mitochondrial membrane potential (ΔΨm) was detected using JC-1, according to the manufacturer's instructions. Each experimental group underwent a minimum of three independent repetitions.

### LDH release assay

LDH levels in cell culture supernatants were quantified by using an assay kit as per the manufacturer's instructions. Supernatant LDH activity is expressed as the percentage of total LDH in the cell lysate.

### Microscopy

To observe morphological changes in apoptotic or pyroptotic cells, cells were seeded in 6 well plates and treated as specified. Bright field images were captured using an optical inverted microscope. The image data presented encompass at least three randomly selected fields of view.

### RNA-seq and Mass Spectrometric Analysis

Total RNA was extracted from mouse kidney tissues by the PureLink® RNA Mini Kit. The BerryGenomics company (Beijing, China) provided the main part of the RNA-seq and bioinformatics analysis. Mass spectrometric analysis was conducted at the Novogene Bioinformatics Institute (Beijing, China).

### Real-time PCR

cDNA was synthesized from total RNA by All-in-One First-Strand Synthesis Master Mix (Yugong Biotech). qRT-PCR was performed with F488 SYBR qPCR Mix (Universal) (EG23111L, BestEnzymes). And 2^(-△△Ct)^ was used to compare the mRNA expression between the affected individuals and the controls. Each assay was performed in five independent tests. The primer sequences are listed in Table S1.

### Chromatin immunoprecipitation (ChIP)

The quantitative ChIP assay was performed by using the Abcam ChIP Kit (ab500, UK). The enriched DNA was measured by real-time PCR as described above using the primers 5'-TCTGCCTCCCAAGTGCT-3' (sense) and 5'-GGAAGGACCCACAGACAGA-3' (antisense) for *p21* promoter. The results were normalized with each histone pull-down Ct value.

### CCK8 and flow cytometric analysis

Cell viability was assessed by a Cell counting Kit-8. Cells were seeded in a 96 well plate with 5000 cells/well. After stimulation, the CCK-8 solution was added to the culture medium, and the cultures were incubated for 1 h at 37 °C in humidified 95% air and 5% CO_2_. The absorbance was measured at 450 nm using a Microplate Reader (Bio-Rad, Hercules, CA).

The PTECs were seeded in 6 well plates and exposed to various concentration of cisplatin for 18 hours. The cells were obtained and resuspended in 195 μl of 1× binding buffer containing Annexin V-FITC (5 μl) and PI (10 μl), and cells were incubated away from light for 20 min. The results were evaluated immediately through flow cytometry (BD FACSCanto, NJ, USA).

### Acridine orange/ethidium bromide (AO/EB) staining and Hoechst staining

AO/EB double staining was performed to evaluate the morphological changes of cells due to apoptosis. PTECs were cultured in 6-well plates and treated with cisplatin for 18 hours. Post-incubation, cells were obtained and resuspended in AO/EB (0.1 mg/mL), placed on slides, and observed under a fluorescent microscope (Leica DM4000B).

Hoechst staining was used to examine the nuclear morphological changes. After treatment as described previously, cells were obtained and resuspended in Hoechst (1 mg/mL in PBS), placed on slides, and observed under a fluorescent microscope (Leica DM4000B).

### Statistical analysis

Data were subjected to statistical analysis with Graph-Pad Prism 5 (GraphPad Software) and plotted by AI Illustrator (Adobe). Results represent the mean ± SEM of at least 3 independent experiments as indicated in the figure legends. Two-tailed Student's t-tests based on ANOVA were used for 2-group comparisons. For qRT-PCR analysis, we performed ΔΔCT methods. Differences were considered statistically significant at P < 0.05, with significance indicated in figures as *p < 0.05, **p < 0.01, ***p < 0.001, ns represents no significant difference.

## Results

### A strong linkage between high expression of Lipin3 and AKI

**Figure 1:**
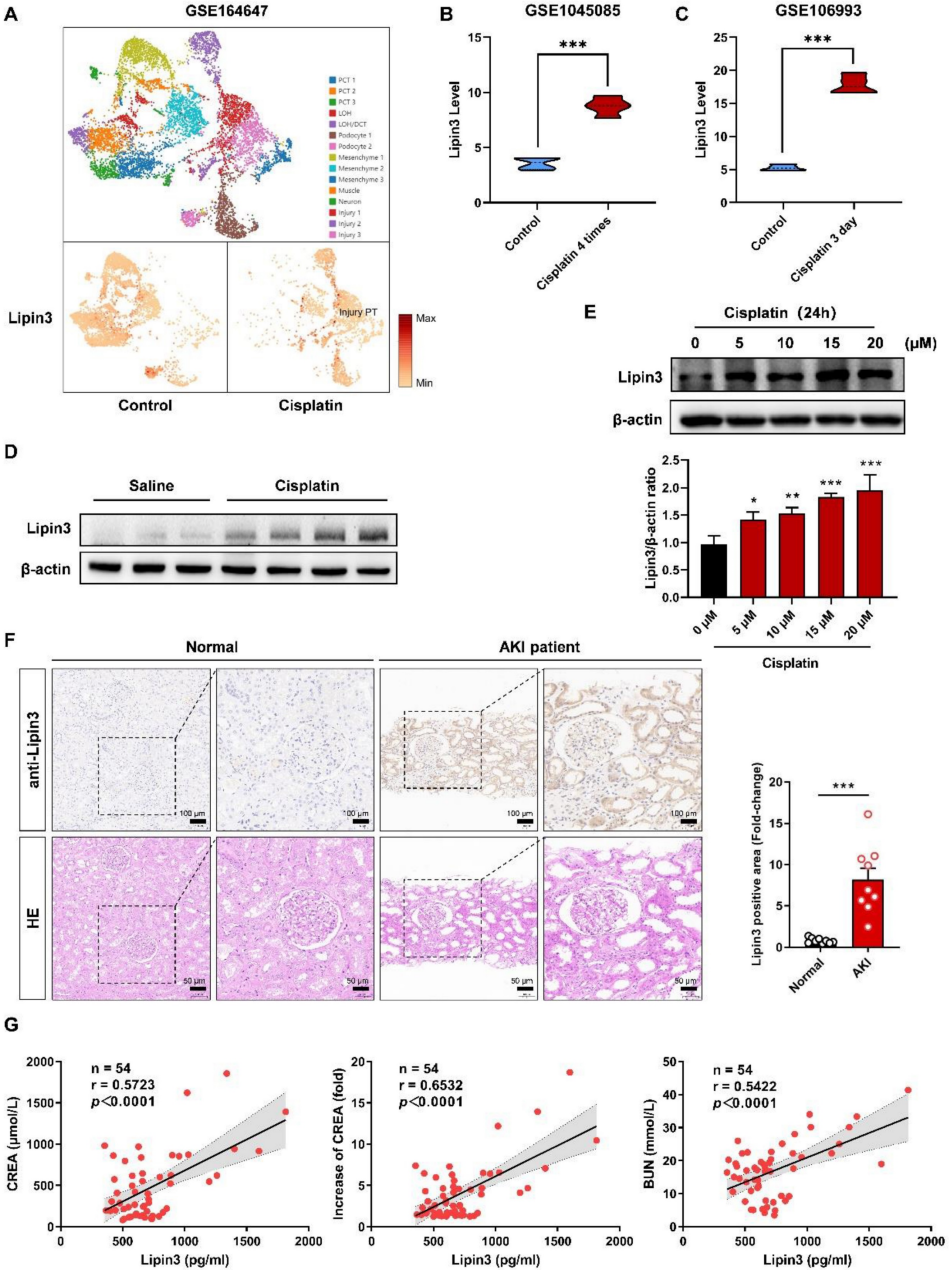
** Link between high expression of Lipin3 and cisplatin-induced AKI.** (A) The distribution and relative expression of *Lipin3* gene in human injured kidney organoids (From snRNA-Seq database, GSE164647). The *Lipin3* gene expression in human kidney organoids (GSE145085 22) (B) and mouse kidneys (GSE106993 21) (C) after cisplatin treatment. (D) WB analysis showing the expression levels of Lipin3 in mice treated with or without cisplatin. (E) WB analysis showing the expression levels of Lipin3 in HK2 cells treated with different concentrations of cisplatin. (F) IHC staining and HE staining showing the representative conditions of Lipin3 expression and renal tubular injury in kidney tissues from healthy control subjects (n = 9) and AKI patients (n = 9). (G) Scatter plots showing the correlation between serum Lipin3 levels and serum creatinine levels (left), the fold increase in serum creatinine (middle) and serum BUN levels (right) in 54 AKI patients. Correlation coefficient r and *p* value were calculated by the Spearman's rank correlation coefficient test.

Because there was no previous study focusing on Lipin3 and kidney disease, we first analyzed the RNA levels of *Lipin3* in public database. The single-cell RNA-seq database (GSE164647) of cisplatin treated human kidney organoids revealed that the mRNA levels of *Lipin3* was significantly increased in injury proximal tubular cells after cisplatin treatment (Figure 1A). The GSE145085 database (four controls and four cisplatin treated human kidney organoids) and GSE106993 database (kidney tissues from four controls and four mice treated with cisplatin) showed that the mRNA levels of *Lipin3* in cisplatin treated group were higher than control group (Figure 1B and C). The expression of the other two members of the Lipin family, Lipin1 and Lipin2, can be seen in Figure S1A-D. We then generated the AKI mouse model by intraperitoneal injecting cisplatin (Figure S2A-C). The WB analysis indicated higher levels of Lipin3 protein in cisplatin-treated mouse kidney tissues compared to the saline group (Figure 1D). Similarly, immunofluorescence staining of mouse kidney sections showed that the expression of Lipin3 in the kidneys was upregulated after cisplatin treatment, and co-localized with both proximal and distal tubules (Figure S3). *In vitro*, we treated HK-2 cells with cisplatin to model the condition and analyzed Lipin3 expression via WB. The results showed that Lipin3 expression was upregulated as early as 1 hour after cisplatin treatment and continued to increase over 24 hours (Figure S4). Moreover, under the same treatment duration, higher concentrations of cisplatin led to greater upregulation of Lipin3 expression (Figure 1E). Finally, we collected kidney biopsy tissues from 9 AKI patients and 9 healthy controls (kidney contusion patients), as well as serum samples from 54 AKI patients (Table S2 and S3). The IHC analysis indicated that the protein levels of Lipin3 were higher in AKI patients compared to healthy controls (Figure 1F and Figure S5). Simultaneously, the serum ELISA detection also supported that the serum Lipin3 levels were positively correlated with serum creatinine levels, the fold increase in serum creatinine and serum Bun levels in AKI patients, particularly in patients with nephrotoxic AKI (Figure 1G and Figure S6). These findings in public databases, patients, mice, and cell lines collectively suggest a strong linkage between high Lipin3 levels and AKI or cisplatin induced AKI.

### Overexpression of Lipin3 alleviated cisplatin induced AKI in mice model

**Figure 2:**
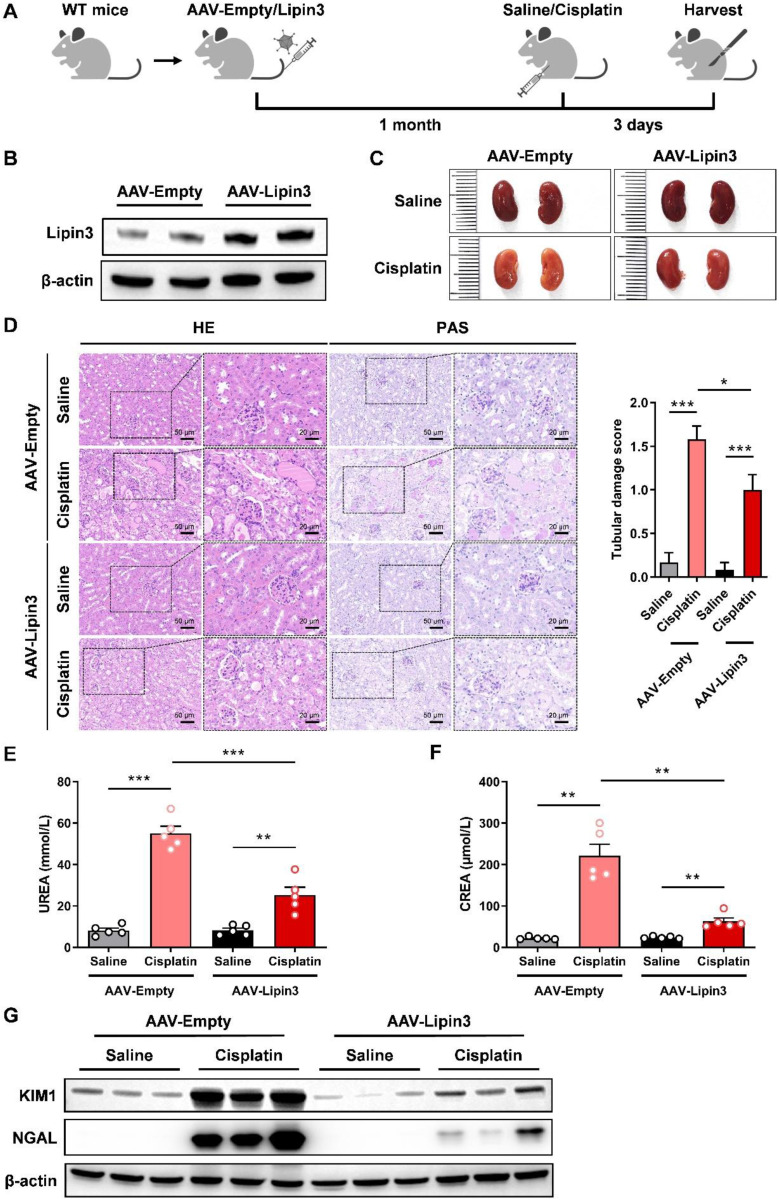
** Overexpression of Lipin3 in mice can alleviate cisplatin-induced AKI.** (A) Flow chart of animal study design. (B) WB analysis confirmed successful overexpression of Lipin3 in the mice kidney. (C) Kidney appearance of AAV-Empty mice (n = 5) and AAV-Lipin3 mice (n = 5) treated with or without cisplatin. (D) HE staining analysis showing the renal tubular injury in AAV-Empty mice (n = 5) and AAV-Lipin3 mice (n = 5) treated with or without cisplatin. Peripheral blood UREA (E) and creatinine (F) levels of AAV-Empty mice (n = 5) and AAV-Lipin3 mice (n = 5) treated with or without cisplatin. (G) WB analysis showed the expression of Lipin3, KIM1 and NGAL in AAV-Empty mice (n = 5) and AAV-Lipin3 mice (n = 5) treated with or without cisplatin.

To investigate the role of Lipin3 upregulation in AKI, we used 8-week-old WT mice and constructed Lipin3 overexpression mice by tail vein injection of AAV virus, and then induced AKI by intraperitoneal injection of cisplatin (Figure 2A and B). After euthanasia, compared to the kidneys of cisplatin-treated AAV-Empty mice, the kidneys of AAV-Lipin3 mice were closer in color to those of saline-injected mice (Figure 2C), indicating that the degree of kidney ischemia in the cisplatin-induced AAV-Lipin3 group was milder than in the AAV-Empty group. HE staining and PAS staining showed that kidney tubular damage was reduced in cisplatin-treated AAV-Lipin3 mice compared to cisplatin-induced AAV-Empty mice (Figure 2D). After cisplatin treatment, serum blood urea nitrogen (BUN) levels and creatinine levels in the AAV-Lipin3 group were lower than in the AAV-Empty group (Figures 2E and F). Additionally, the protein levels of kidney injury markers KIM and NGAL in the renal tissue of AAV-Lipin3 mice were significantly lower than those in WT mice (Figure 2G). These results suggest that the upregulation of Lipin3 has a protective effect in cisplatin-induced AKI.

### Lipin3 deficiency aggravated cisplatin induced AKI in mice model

**Figure 3:**
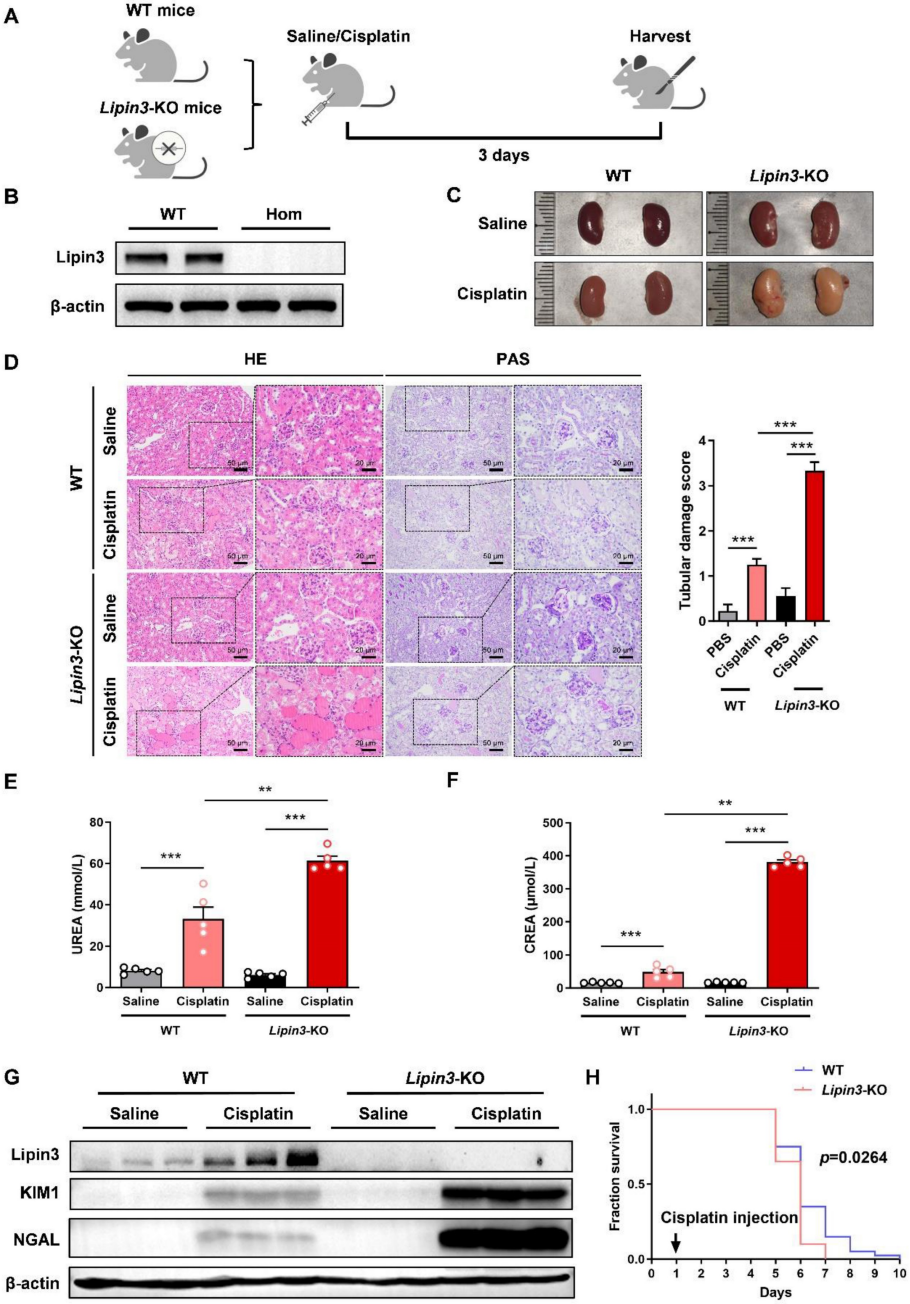
** Lipin3-KO mice were more sensitive to cisplatin-induced AKI.** (A) Flow chart of animal study design. (B) WB analysis verified the successful generation of Lipin3-KO mice. (C) Kidney appearance of WT mice (n = 5) and *Lipin3*-KO mice (n = 5) treated with or without cisplatin. (D) HE staining analysis showing the renal tubular injury in WT mice (n = 5) and *Lipin3*-KO mice (n = 5) treated with or without cisplatin. Peripheral blood UREA (E) and creatinine (F) levels of WT mice (n = 5) and *Lipin3*-KO mice (n = 5) treated with or without cisplatin. (G) WB analysis showed the expression of Lipin3, KIM1 and NGAL in WT mice (n = 5) and Lipin3-KO mice (n = 5) treated with or without cisplatin. (H) Survival curve of each group (n=40).

To further reveal the relationship between Lipin3 and AKI, we generated *Lipin3*-KO mice (Figure S7A and B). Then, we selected *Lipin3*-KO mice (n = 5) and WT control littermates (n = 5) aged 8 weeks to establish AKI mice model by cisplatin intraperitoneal injection (Figure 3A and B). After euthanasia treatment, the kidney of *Lipin3*-KO mice presented with pale color compared to WT mice after cisplatin treatment (Figure 3C), which indicated the more severe renal ischemia in cisplatin induced *Lipin3*-KO group than in WT group. Analysis of the body weight of mice before and after cisplatin injection revealed that the weight loss in *Lipin3*-KO mice after cisplatin injection was significantly higher than that in WT mice (Figure S7C). HE staining and PAS staining revealed that compared to cisplatin induced WT mice, the *Lipin3*-KO mice showed more serious renal tubular injury after cisplatin treatment (Figure 3D). The serum BUN levels and creatinine levels were increased more dramatically in *Lipin3*-KO group than WT group after cisplatin treatment (Figure 3E and F). Simultaneously, protein levels of kidney injury marker, KIM and NGAL, were also increased more significantly in *Lipin3*-KO group than WT group after cisplatin treatment (Figure 3G), and the mRNA levels of *CTR1* and *OCT2*, two molecules affecting the uptake and cytotoxicity of platinum drugs,^28^,^29^ showed the opposite tendency (Figure S7D and E). Finally, we selected 40 WT and *Lipin3*-KO mice each to inject the cisplatin and continuous feeding to analyze the lifetime, the results showed that the lifetime of *Lipin3*-KO group were shorter than WT group (Figure 3H). These observations suggested that the deficiency of Lipin3 may aggravate the process of cisplatin induced AKI.

### Lipin3 deficiency exacerbated cisplatin induced mitochondrial dysfunction

**Figure 4:**
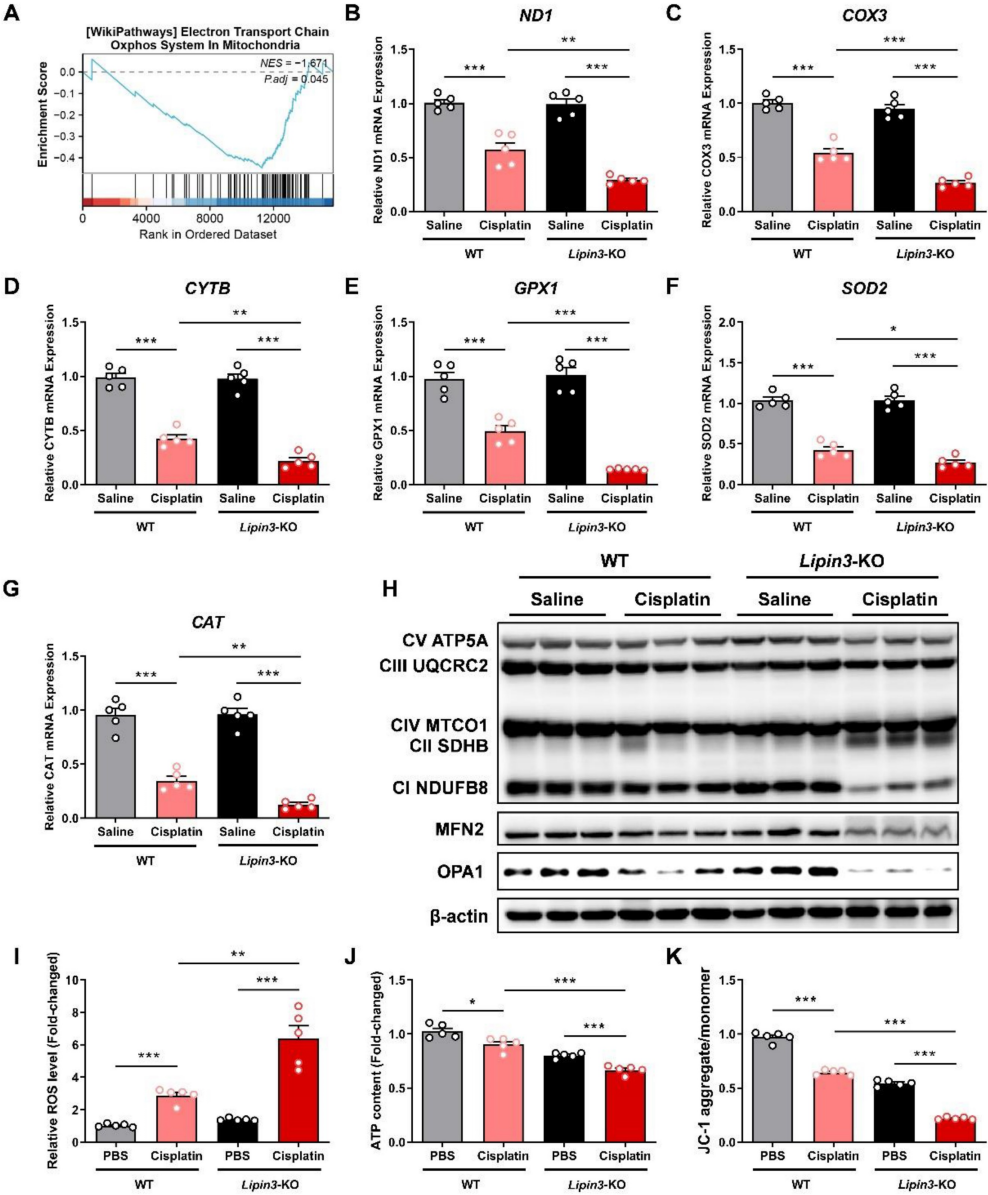
** Lipin3 deficiency exacerbated cisplatin induced mitochondrial dysfunction.** (A) GSEA analysis for KEGG enrichment in the RNA-seq data. mRNA levels of *ND1* (B), *COX3* (C), *CYTB* (D), *GPX1* (E), *SOD2* (F) and *CAT* (G) in the kidney of mice in each group. (H) WB analysis revealed the expressions of mitochondrial electronic respiratory chain-related proteins, MFN2 and OPA1 in the kidney of WT mice and *Lipin3*-KO mice treated with or without cisplatin. ROS levels (I), ATP levels (J) and JC-1 staining (K) in WT and *Lipin3*-KO PTECs treated with or without cisplatin.

Mitochondrial function stands as one of the crucial indicators for monitoring the progression of AKI. Therefore, we further conducted mitochondrial-related assessments on each group. The kidney tissues of WT and *Lipin3*-KO mice after cisplatin treatment were used to analyze the potential mechanisms between Lipin3 and AKI by RNA-seq. GSEA based on DEGs between the WT and *Lipin3*-KO mice after cisplatin treatment groups revealed that “Oxidative phosphorylation” was significantly enriched with KEGG analysis (Figure 4A). Interestingly, we found the mRNA levels of three genes, *ND1*, *COX3* and *CYTB* were decreased dramatically in cisplatin induced *Lipin3*-KO group, which were further confirmed by real-time PCR (Figure 4B-D). These three genes, *ND1*, *COX3* and *CYTB*, are mtDNA markers, which are used to assess the number of mitochondria.^30^,^31^ Moreover, some anti-oxidases including *GPX1*, *SOD2* and *CAT*^32^,^33^ were also reduced in cisplatin induced *Lipin3*-KO group (Figure 4E-G). Biochemical assays revealed that the total antioxidant capacity (TAOC) in the kidney tissues of *Lipin3*-KO mice treated with cisplatin was lower than that in WT mice (Figure S8A). WB analysis revealed that after cisplatin treatment, the expression levels of two crucial components of the mitochondrial respiratory chain,^34^ NDUFB8 and SDHB, were significantly altered in Lipin3-KO mice compared to WT mice (Figure 4H). Moreover, the expression of MFN2 and OPA1, two proteins maintaining the structure and function of the mitochondria 35,36, were also decreased in cisplatin induced *Lipin3*-KO group (Figure 4H). Immunofluorescence results revealed that after cisplatin treatment, the levels of ROS in the kidney tissues of *Lipin3*-KO mice were significantly higher than those in WT mice (Figure S8D). We then isolated the PTECs from WT and *Lipin3*-KO mice. The ROS levels in cisplatin induced *Lipin3*-KO group were increased dramatically (Figure 4I), and the ATP levels were reduced (Figure 4J). The JC-1staining indicated that the mitochondrial membrane potential in cisplatin induced *Lipin3*-KO group was decreased obviously (Figure 4K). The data relating to mitochondria indicated that the Lipin3 deficiency can exacerbate cisplatin induced mitochondrial dysfunction.

### Lipin3 deficiency activate the apoptosis and Caspase 3/GSDME‑dependent secondary pyroptosis in cisplatin induced AKI

**Figure 5:**
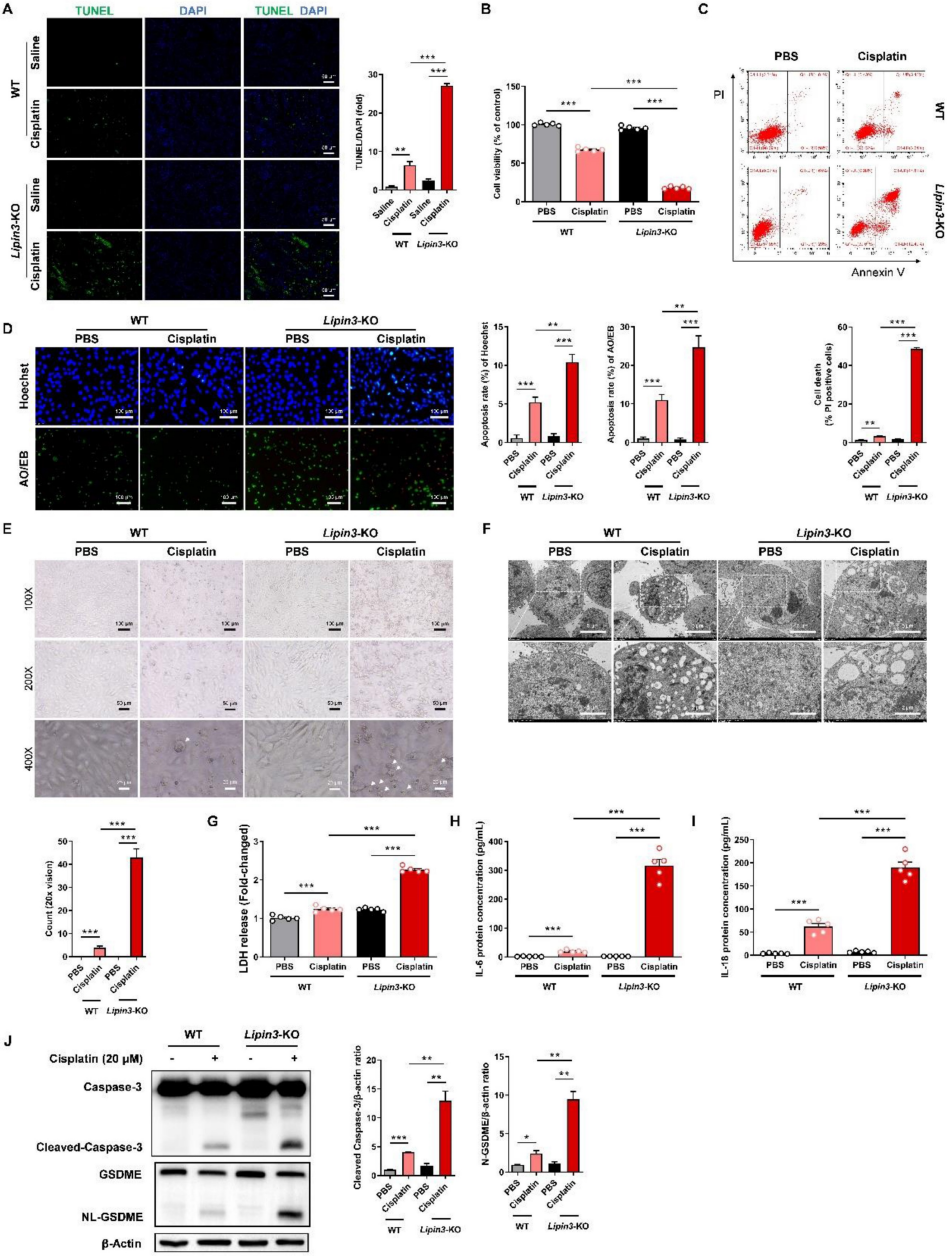
**Lipin3 deficiency activate the apoptosis and Caspase 3/GSDME‑dependent secondary pyroptosis in cisplatin induced AKI.** (A) The TUNEL staining analysis showing the apoptosis in the kidney of WT mice (n = 5) and Lipin3-KO mice (n = 5) treated with or without cisplatin. The fluorescence intensity in the graphs was calculated using Image J software analysis. (B) CCK8 analysis showing the cell viability of WT and *Lipin3*-KO PTECs treated with or without cisplatin. (C) Flow cytometric analysis of PI- and Annexin V-FITC-stained PTECs treated with or without cisplatin. (D) Hoechst staining (top), AO/EB staining (bottom), and corresponding statistical analysis of apoptosis rates for each group of cells. (E) Phase-contrast images and (F) TEM analysis of WT and *Lipin3*-KO PTECs were examined after cisplatin treatment. Arrows, pyroptotic cells. The levels of LDH (G), IL-6 (H) and IL-18 (I) in cell supernatant were measured. (J) WB analysis revealed the expressions of Caspase 3, Cleaved-Caspase 3, GSDME, N-GSDME in WT and *Lipin3*-KO PTECs treated with or without cisplatin.

Mitochondrial dysfunction can further active the mitochondrial apoptotic pathway, resulting in cellular death. Consequently, we proceeded to investigate the apoptosis condition in AKI models with Lipin3 deficiency. The TUNEL staining of mice kidney tissues indicated that the number of apoptotic cells in Lipin3-KO mice was much higher than WT mice after cisplatin treatment (Figure 5A). *In vitro*, the CCK-8 assay results revealed that, following cisplatin stimulation for 18 hours, the viability of *Lipin3*-KO PTECs decreased proportionally with the increasing concentration of cisplatin (Figure S9A). As the cisplatin treatment duration extended to 24 hours, the viability of PTECs in *Lipin3*-KO group further declined significantly compared to the WT group (Figure 5B and Figure S9B). The flow cytometric analysis with Annexin V-FITC and PI staining suggested that after 18 hours of cisplatin stimulation (20μM), the number of apoptotic PTECs in the *Lipin3*-KO group was markedly higher than that in the WT group (Figure S9C). Similarly, with an increase in cisplatin treatment duration to 24 hours, the disparity between the WT and *Lipin3*-KO groups widened (Figure 5C and Figure S9D). Both Hoechst and AO/EB staining results indicated that after cisplatin treatment, the apoptotic rate in the *Lipin3*-KO group was significantly higher compared to the WT group (Figure 5D). During primary cell culture and cisplatin treatment, light microscope observation showed more typical swollen cells in cisplatin induced *Lipin3*-KO group than in cisplatin induced WT group (Figure 5E), electron microscopy observations revealed obvious pores in the cell membrane and severe swelling of cellular organelles (Figure 5F), which indicated that deletion of Lipin3 may also activate the pyroptosis in cisplatin treated PTECs.^37^,^38^ LDH release, IL-6 and IL-18 secretion in cell supernatant were all increased in cisplatin treated *Lipin3*-KO group, compared to cisplatin treated WT group (Figure 5G-I). The *in vivo* findings mirrored those observed in the *in vitro* experiments (Figure S10A-C). WB analysis further confirmed that the protein levels of activated Caspase 3 and cleaved GSDME were increased dramatically in cisplatin induced *Lipin3*-KO group (Figure 5J and Figure S11). These results suggested that the Lipin3 deficiency can activate cell apoptosis and Caspase 3/GSDME‑dependent secondary pyroptosis in cisplatin induced PTECs.

### Inhibition of GSDME-mediated pyroptosis pathway could rescue AKI aggravated by Lipin3 deficiency

**Figure 6:**
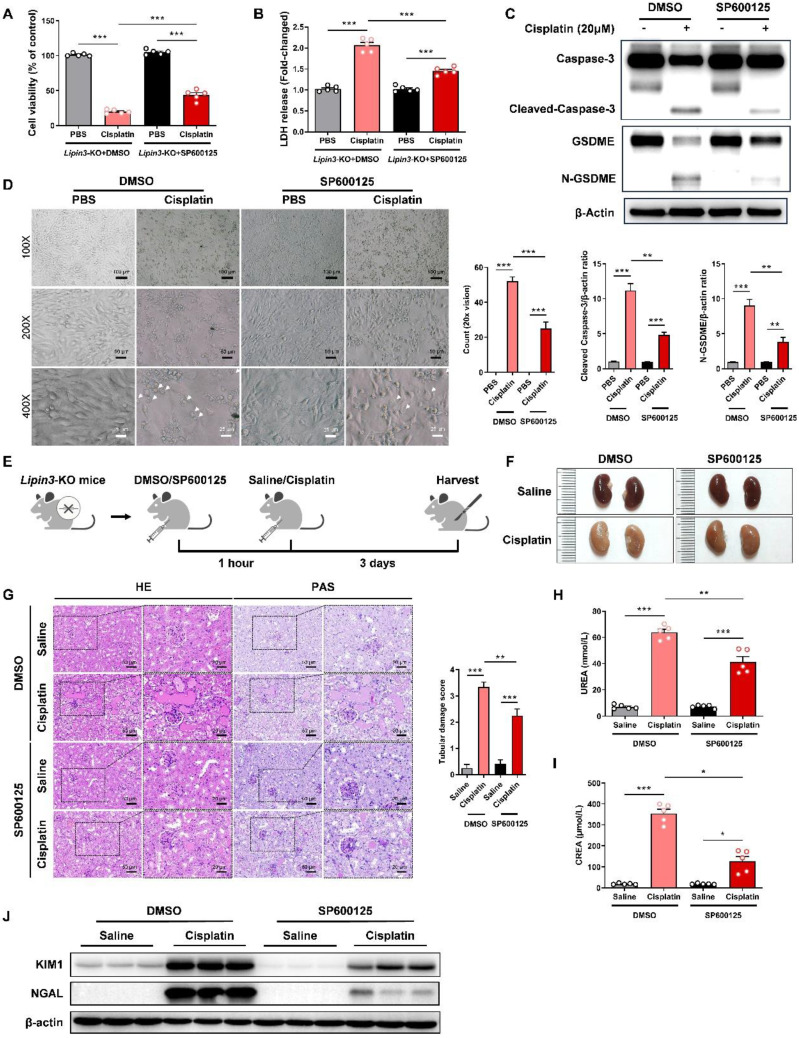
** SP600125 alleviate cisplatin-induced renal pyroptosis *in vivo*.** (A) CCK8 analysis showing the cell viability in *Lipin3*-KO PTECs treated with DMSO or SP600125 for 1 h, followed by treating with or without cisplatin. (B) The levels of LDH in *Lipin3*-KO PTECs treated with DMSO or SP600125 for 1 h, followed by treating with or without cisplatin were measured. (C) WB analysis revealed the expressions of Caspase 3, Cleaved-Caspase 3, GSDME, N-GSDME in *Lipin3*-KO PTECs treated with DMSO or SP600125 for 1 h, followed by treating with or without cisplatin. (D) Phase-contrast images of *Lipin3*-KO PTECs treated with DMSO or SP600125 for 1 h, followed by treating with or without cisplatin. Arrows, pyroptotic cells. (E) Flow chart of animal study design. (F) Kidney appearance of cisplatin-treated* Lipin3*-KO mice (n = 5) with or without SP600125 pretreatment. (G) HE staining analysis showing the renal tubular injury in cisplatin-treated* Lipin3*-KO mice (n = 5) with or without SP600125 pretreatment. Peripheral blood UREA (H) and creatinine (I) levels of cisplatin-treated* Lipin3*-KO mice (n = 5) with or without SP600125 pretreatment. (J) WB analysis showed the expression of KIM1 and NGAL in cisplatin-treated *Lipin3*-KO mice (n = 5) with or without SP600125 pretreatment.

Earlier studies have demonstrated that the JNK inhibitor SP600125 can effectively prevent GSDME-mediated pyroptosis.^39^,^40^ Building on these findings, we proceeded to investigate the impact of SP600125 on pyroptosis exacerbated by Lipin3 deficiency. To do this, we selected *Lipin3*-KO PTECs and pre-treated them with SP600125 prior to cisplatin exposure. The results indicated that compared to cisplatin-treated Lipin3-KO PTECs, those pretreated with SP600125 before cisplatin treatment exhibited significantly improved cell viability (Figure 6A), reduced LDH release (Figure 6B). WB analysis revealed that the levels of cleaved-Caspase 3 and N-GSDME in *Lipin3*-KO PTECs pretreated with SP600125 and subsequently treated with cisplatin were lower than those in *Lipin3*-KO PTECs treated with cisplatin alone (Figure 6C). Additionally, under light microscopy, it was found that compared to Lipin3-KO PTECs treated with cisplatin only, the number of pyroptotic cells was markedly decreased following pretreatment with SP600125 (Figure 6D). In our *in vivo* experimental studies, we utilized 8-week-old *Lipin3*-KO mice. One hour prior to the induction of AKI with cisplatin, the mice were intraperitoneally injected with either SP600125 or a vehicle control (Figure 6E). Our findings revealed that SP600125 significantly alleviated renal ischemia and renal tubular injury induced by cisplatin in *Lipin3*-KO mice (Figure 6F and G). SP600125 also suppressed the increased serum BUN and creatinine induced by cisplatin in *Lipin3*-KO mice (Figure 6H and I). Meanwhile, the protein levels of KIM and NGAL decreased following SP600125 pretreatment (Figure 6J). These results indicated that inhibition of GSDME-mediated pyroptosis pathway by SP600125 could rescue AKI aggravated by Lipin3 deficiency.

### Lipin3 can regulate the Caspase 3/GSDME axis through p21

**Figure 7:**
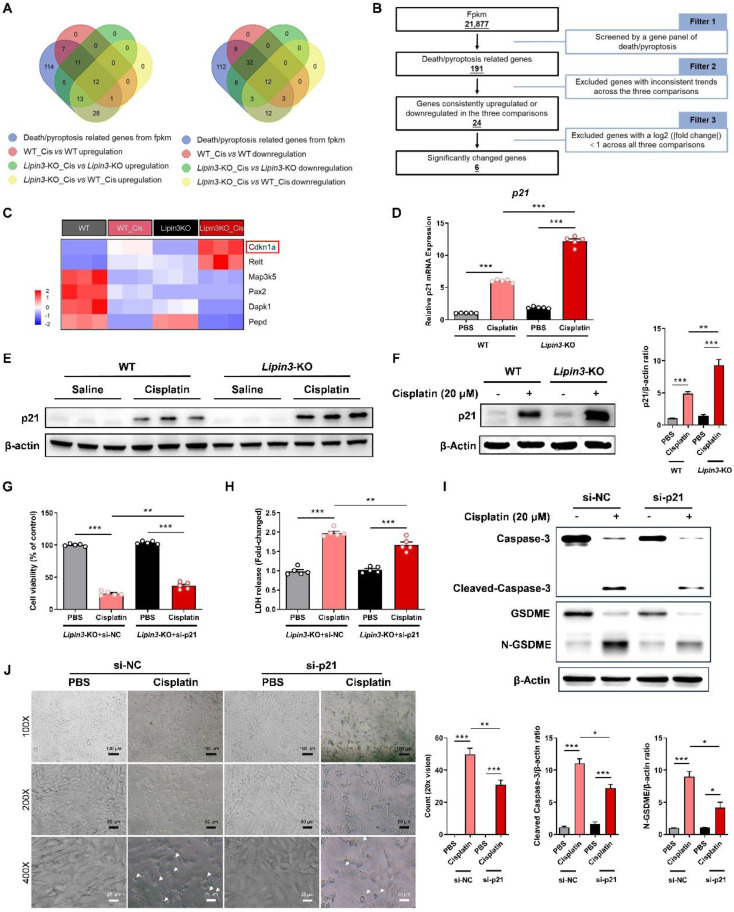
** Lipin3 can regulate the Caspase 3/GSDME axis through p21.** (A)The Venn analysis provided a schematic representation highlighting the overlap of upregulated (left) and downregulated (right) death/pyroptosis-related genes. (B) Schematic of the filter strategies used in our study. (C) Heat maps illustrate the expression patterns of the selected genes across various mouse groups. (D) mRNA levels of *p21* in WT and *Lipin3*-KO PTECs treated with or without cisplatin. (E) WB analysis revealed the expressions of p21 in the kidney of WT mice (n = 5) and *Lipin3*-KO mice (n = 5) treated with or without cisplatin. (F) WB analysis revealed the expression of p21 in WT and *Lipin3*-KO PTECs treated with or without cisplatin. (G) CCK8 analysis showing the cell viability in *Lipin3*-KO PTECs transfected with scrambled or *p21* siRNA followed by treating with or without cisplatin. (H) The levels of LDH in *Lipin3*-KO PTECs transfected with scrambled or *p21* siRNA followed by treating with or without cisplatin were measured. (I) WB analysis revealed the expressions of Caspase 3, Cleaved-Caspase 3, GSDME, N-GSDME in *Lipin3*-KO PTECs transfected with scrambled or *p21* siRNA followed by treating with or without cisplatin. (J) Phase-contrast images of *Lipin3*-KO PTECs transfected with scrambled or *p21* siRNA followed by treating with or without cisplatin. Arrows, pyroptotic cells.

We confirmed that Lipin3 deficiency may aggravated cisplatin induced AKI by affecting mitochondria and activating apoptosis and Caspase 3/GSDME‑dependent secondary pyroptosis in PTECs.^41^-^43^ However, it was not clear how Lipin3 regulated mitochondria and cell death. The kidney tissues of WT and Lipin3-KO mice after cisplatin treatment were used to analyze the potential mechanisms between Lipin3 and AKI by RNA-seq. We performed a comprehensive analysis of RNA sequencing data to identify differentially expressed genes (DEGs) across three comparisons: WT cisplatin-treated vs. WT control, Lipin3-KO cisplatin-treated vs. Lipin3-KO control, and Lipin3-KO cisplatin-treated vs. WT cisplatin-treated. We focused on genes consistently upregulated or downregulated in these comparisons and intersected them with a set of genes linked to cell death/necroptosis. This process led to the identification of 12 commonly upregulated and 12 commonly downregulated DEGs associated with cell death/pyroptosis (Figure 7A). After excluding genes with a log2 (|fold change|) less than 1 across all three comparisons, we narrowed down the list to 6 significant DEGs (Figure 7B).

After literature search, we ultimately identified the significantly DEG, Cyclin Dependent Kinase Inhibitor 1A (*CDKN1A*) as potentially involved in the regulation of our mechanism (Figure 7C). CDKN1A, also called p21, is an important biomarker of AKI and renal aging.^44^,^45^ Previous studies have shown that p21 upregulation can promote the Caspase 3 activation and ROS generation, which further induce cell cycle arrest, apoptosis, and Caspase 3/GSDME‑dependent secondary pyroptosis.^46^,^47^ Multiple factors including renal artery stenosis, ischemia-reperfusion injury and Aflatoxin B1 have been explored by upregulating p21 to induce kidney injury and renal toxicity.^48^,^49^ Real-time PCR and WB analyses revealed that the expression of p21 in the kidney tissues of cisplatin-treated *Lipin3*-KO mice was significantly increased compared to that in cisplatin-treated WT mice (Figure 7D and E). *In vitro* experiments, the expression of p21 in PTECs treated with cisplatin was consistent with the trend observed in the kidney tissues from *in vivo* experiments (Figure 7F). In addition, we selected the *Lipin3*-KO PTECs and transfected them with p21 siRNA (Figure S12A and B) before cisplatin treatment. Our study results indicated that compared to cisplatin-induced *Lipin3*-KO PTECs, *Lipin3*-KO PTECs treated with cisplatin and transfected with p21 siRNA exhibited increased cell viability, reduced LDH release in the cell supernatant (Figure 7G and H). WB analysis revealed decreased levels of active Caspase 3 and cleaved GSDME in p21-siRNA-transfected PTECs treated with cisplatin versus Lipin3-KO PTECs under the same treatment (Figure 7I).

Additionally, under light microscopy, it was found that compared to *Lipin3*-KO PTECs treated with cisplatin only, the number of pyroptotic cells was markedly decreased following transfection with p21 siRNA (Figure 7J). Hence, we hypothesize that Lipin3 deficiency may activate the p21-Caspase 3 pathway and affect the mitochondria and cell death.

### Lipin3 can regulate the p21-Caspase 3/GSDME pathway by interacting with Sirt1

**Figure 8:**
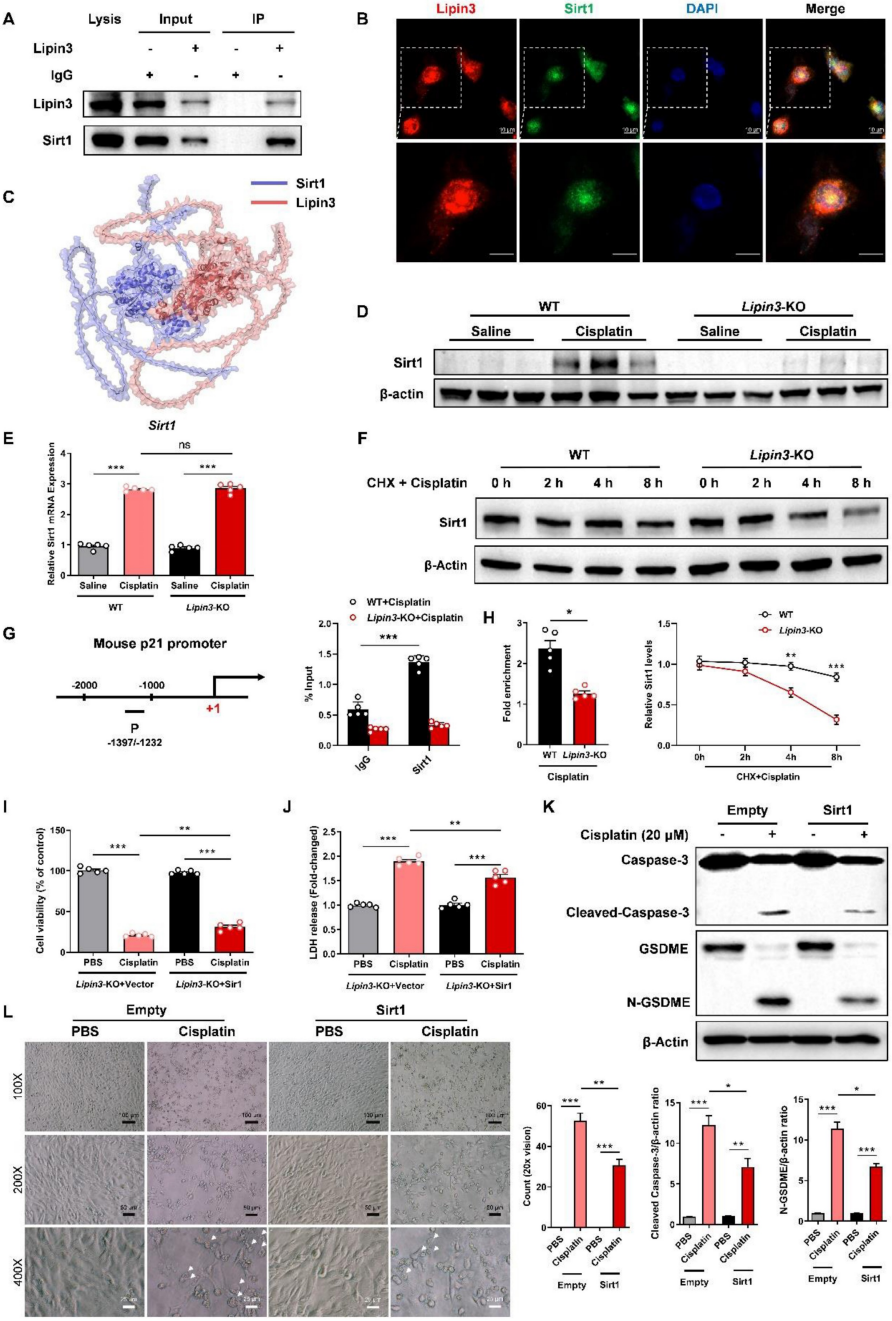
** Lipin3 can regulate the p21-Caspase 3/GSDME pathway by interacting with Sirt1.** (A) Co-IP analysis confirmed the interaction between Lipin3 and Sirt1 in HK2 cells. (B) Immunofluorescence staining showing the subcellular localization of Lipin3 and Sirt1 in HK2 cells. (C) Molecular docking results indicate that Sirt1 is a potential interacting protein of Lipin3. (D) WB analysis revealed the expressions of Sirt1 in the kidney of WT mice (n = 5) and *Lipin3*-KO mice (n = 5) treated with or without cisplatin. (E) mRNA levels of *Sirt1* in WT and *Lipin3*-KO PTECs treated with or without cisplatin. (F) WB analysis revealed the degradation rate of Sirt1 in WT and *Lipin3*-KO PTECs. (G) ChIP assays were performed in WT and *Lipin3*-KO PTECs using Sirt1-specific antibody. ChIP-qPCR showed the binding of Sirt1 protein to the p21 gene promoter in WT and Lipin3KO cells treated with cisplatin. IgG was used as a negative control. (H) Enrichment of *p21* promoter was measured by qPCR. (I) CCK8 analysis showing the cell viability in *Lipin3*-KO PTECs transfected with Empty or Sirt1 followed by treating with or without cisplatin. (J) The levels of LDH in *Lipin3*-KO prim PTECs ary cells transfected with Empty or Sirt1 followed by treating with or without cisplatin were measured. (K) WB analysis revealed the expressions of Caspase 3, Cleaved-Caspase 3, GSDME, N-GSDME in *Lipin3*-KO PTECs transfected with Empty or Sirt1 followed by treating with or without cisplatin. (L) Phase-contrast images of *Lipin3*-KO PTECs transfected with Empty or Sirt1 followed by treating with or without cisplatin. Arrows, pyroptotic cells.

However, how can Lipin3 regulate the p21-Caspase 3 pathway? We extracted total proteins from HK2 cells to perform co-IP and mass spectrometric analysis. Our results identified Sirt1 as a novel interacting protein of Lipin3. Additionally, we confirmed the interaction between the two proteins through co-IP validation (Figure 8A). Immunofluorescence staining suggested that the Lipin3 and Sirt1 can co-localized in both cell nucleus and cytoplasm (Figure 8B and Figure S13). Bioinformatics prediction analysis revealed multiple binding sites between Sirt1 and Lipin3 (Figure 8C and Figure S14). The Sirt1, an NAD-dependent protein deacetylase, serves as a transcriptional regulator involved in coordinating various cellular functions like senescence, cell cycle regulation, metabolism, apoptosis, and autophagy.^50^-^52^ Previous study revealed that Sirt1 can repress the expression of p21 through the transcriptional inactivation of p21 promoters.^53^-^55^ The WB results indicated that cisplatin stimulation significantly enhanced the expression of Sirt1 protein in mouse kidney tissues. However, this upregulation was no longer present in *Lipin3*-KO mice (Figure 8D). *In vitro* experiments showed that after cisplatin treatment, the expression of Sirt1 protein in PTECs was also upregulated and increased with prolonged treatment duration. But in Lipin3-KO PTECs, there was a defect in the upregulation of Sirt1 after cisplatin treatment, with a significantly lower increase compared to WT PTECs (Figure S11 and Figure S15A). By detecting the mRNA levels of Sirt1 in cells from each group using qPCR, we found no significant difference in Sirt1 mRNA levels between untreated WT and Lipin3-KO cells. After cisplatin treatment, the mRNA levels of Sirt1 in both cell groups were significantly upregulated, with no significant difference in the extent of upregulation between the two groups (Figure 8E). These results suggested that the upregulation defect of Sirt1 protein was not caused by differences at the transcriptional level. Further examination of the basal expression levels of Sirt1 protein in both cell groups revealed, through WB, that the expression level of Sirt1 protein in *Lipin3*-KO cells was lower than in WT cells, with a statistically significant difference (Figure S15B). Using CHX, a protein synthesis inhibitor, we examined the half-life of Sirt1 protein in both groups of cells treated with cisplatin and found that the half-life of Sirt1 protein in *Lipin3*-KO cells was shorter than in WT cells (Figure 8F). We then assessed the interaction between Sirt1 and the promoter of p21 by ChIP-qPCR study in cisplatin treated WT and *Lipin3*-KO PTECs. The results showed that Sirt1 can bind to the promoter of p21 (Figure 8G). In addition, the interaction between Sirt1 and the promoter of p21 was reduced in cisplatin treated *Lipin3*-KO primary cells compared to WT cells (Figure 8H). These results indicated that under cisplatin treatment, when Lipin3 was deficient, Sirt1 protein was unstable and its abundance decreased, leading to a reduced inhibitory effect on p21 transcription. Subsequently, we conducted rescue experiments with exogenous overexpression of Sirt1 *in vitro* to validate this finding. We opted for *Lipin3*-KO PTECs and transfected them with Sirt1 eukaryotic expression plasmids (Figure S16A and B) prior to cisplatin treatment. Our study results demonstrated that, compared to Lipin3-KO cells transfected with an empty vector, those transfected with a Sirt1 plasmid and subsequently treated with cisplatin exhibited significantly increased cell viability (Figure 8I), reduced LDH release in the cell supernatant (Figure 8J), decreased expression of activated Caspase 3 and cleaved GSDME (Figure 8K), and notably, a marked decrease in the number of pyroptotic cell (Figure 8L). These findings indicated that Lipin3 can interact with Sirt1, maintaining the stability of Sirt1 protein and preserving Sirt1's ability to repress p21 transcription.

**Figure 9:**
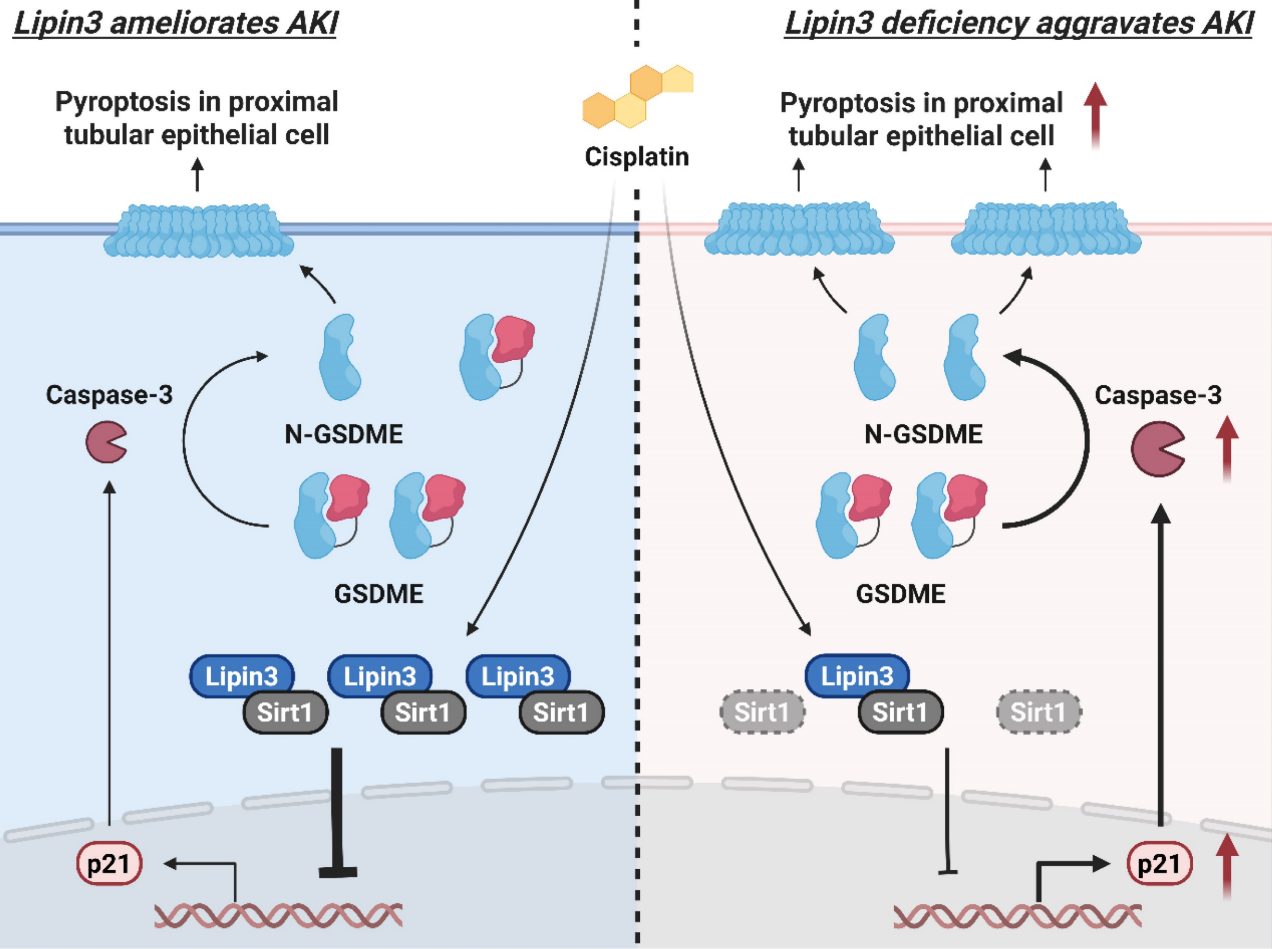
The mechanism by which Lipin3 regulates the Sirt1-p21-Caspase 3-GSDME pyroptosis signaling pathway in AKI.

Collectively, Lipin3 deficiency activates apoptosis, and Caspase 3/GSDME-dependent secondary pyroptosis may occur via the Sirt1-p21-Caspase 3 pathway. The mechanism study revealed that Lipin3 can interact with Sirt1 to maintain the stability of Sirt1 protein. Sirt1 can further suppress the p21 promoter, inhibiting the p21-caspase 3 pathway. After cisplatin treatment, the absence of Lipin3 leads to decreased stability and reduced abundance of Sirt1 protein, thereby weakening its ability to inhibit p21, resulting in overexpression of p21 and further activation of Caspase 3 and GSDME. This ultimately leads to mitochondrial dysfunction, apoptosis, and Caspase 3/GSDME-dependent secondary pyroptosis in PTECs, exacerbating cisplatin-induced AKI (Figure 9).

## Discussion

At present, there were several studies focused on exploring the pathophysiological roles of Lipin3. For example, deficiency in Lipin2 and Lipin3 can elevate plasma triglyceride levels and reduce weight by activating the mTORC1 pathway and modulating chylomicron synthesis.^56^ Lipin1 and lipin3 collectively determine adiposity *in vivo*, and the absence of Lipin3 might compromise the adipogenic capacity of stromal vascular cells.^57^ Phosphorylation did not affect the catalytic activity of Lipin3 or its ability to associate with phosphatidic acid *in vitro*.^58^ In addition, several studies indicated that mutations in Lipin3 can result in rhabdomyolysis.^59^-^61^ While the potential involvement of Lipin3 in kidney health and disease has not been previously reported. In this study, we revealed that Lipin3 deficiency in kidney tissue may aggravate cisplatin induced AKI. The analysis of public database, AKI patients and cisplatin treated mice and HK-2 cells suggest a strong linkage between high expression of Lipin3 and AKI. To our knowledge, this study is the first to discuss the role of Lipin3 in kidney diseases. Prior to this, research on Lipin3 and the Lipin family in the field of kidney diseases was scarce, and our research fills this gap. Similarly, each member of the Lipin family is highly expressed in kidney tissue, making the study of their roles in kidney diseases equally promising and valuable.

The relationship between increased Lipin3 and AKI was confirmed in public database, AKI patients and cisplatin treated mice and HK-2 cells. However, in mechanistic study, we found that the deletion of Lipin3 may aggravate cisplatin induced AKI via Sirt1-p21-Caspase 3-GSDME pathway. We supposed that the Lipin3 play a protective effect in the process of AKI. When Lipin3 decreased, the severity of AKI may increase. In AKI patients, there may be a potential feedback mechanism to increase the expression of Lipin3 to inhibit the exacerbation of AKI. This phenomenon was existed in AKI that one protective molecule was increased in diseases but knockdown it may aggravate the process of disease. For example, the Annexin A2 and Tenascin-C were increased in AKI patients and animals, and knockdown the expression of related genes may further deteriorates kidney injury in AKI mice and cells model.^62^,^63^ Hence, our study confirmed the protective effect in the process of AKI and provide a new potential therapeutic target for AKI.

The mechanism by which Lipin3 deficiency aggravates cisplatin induced AKI merits further exploration. In this study, we discovered Sirt1 as a novel Lipin3-interacting protein. Without Lipin3, the stability of Sirt1 decreases, and it cannot be upregulated, thereby potentially reducing its ability to suppress the transcriptional activity of p21. However, regarding how Lipin3 deficiency leads to the instability and degradation of Sirt1 protein, we currently do not have a definitive conclusion, and related research is underway. Once the research is completed, we will organize and publish the findings as our subsequent research output. Previous studies have shown that the Sirt1 can repress the expression of p21 through the transcriptional inactivation of p21 promoters in breast cancer cells, vascular smooth muscle cells and human gingival fibroblasts.^53^-^55^ The overexpression of p21 can lead to cell cycle arrest and activate Caspase 3, which in turn can mediate apoptosis. In AKI condition, apoptotic cells that remain uncleared in the pathological microenvironment undergo secondary lysis and inflammation.

During this phase, Caspase 3 can cleave GSDME, triggering secondary pyroptosis.^42^,^46^,^47^ In our study, the overexpression of p21 can activate the Caspase 3 and GSDME, finally resulting in mitochondrial dysfunction, apoptosis and Caspase 3/GSDME‑dependent secondary pyroptosis in PTECs and aggravated cisplatin induced AKI. In addition, the cell cycle arrest caused by p21 increasing may be a major contributor to accelerate progression of kidney disease.^48^,^49^ Hence, we may also first establish the relationship between Lipin3 and mitochondrial dysfunction, apoptosis and Caspase 3/GSDME‑dependent secondary pyroptosis in AKI model.

Apoptosis of tubular epithelial cells in AKI has been observed in both preclinical models and certain clinical samples. This apoptosis is triggered by both extrinsic and intrinsic pathways, along with ER stress.^11^,^64^,^65^ The pivotal focal points in these pathways leading to apoptosis are the mitochondria, which undergo fragmentation and become sensitized to membrane permeabilization in response to cellular stress. This process results in the release of cell death-inducing factors, such as cytochrome C which can further activate the Caspase 3.^66^,^67^ In our study, the mitochondrial dysfunction was also enhanced in the Lipin3 deficiency PTECs after cisplatin treatment, which further confirmed that mitochondrial dysfunction was the crucial contributor to aggravate AKI. However, the precise mechanisms by which Lipin3 deficiency leads to mitochondrial dysfunction warrant further investigation. Our study found a new gene that can regulate the stability of mitochondria in PTECs.

In recent years, numerous studies have increasingly indicated that pyroptosis was the major contributor to exacerbate the process of AKI.^68^ In cisplatin induced PTECs, upregulation of GSDME-N expression can enhance the release of IL-1β and LDH and decrease the cell viability. Silencing GSDME in mice can attenuate AKI and inflammation.^69^ Autophagy induced GSDME-mediated pyroptosis is the major cause of cobalt chloride induced hypoxia-reoxygenation injury and AKI.^70^ In addition, GSDMD-mediated pyroptosis was widely activated in multiply types of kidney diseases including AKI, diabetic kidney disease, crystal-induced kidney disease and allograft injury in kidney transplantation.^71^,^72^ In our study, the GSDME-mediated pyroptosis but not GSDMD was activated in Lipin3 deficiency PTECs after cisplatin treatment. This activation was induced by the overexpression of p21, an important cell cycle regulator that can induce cell cycle arrest, apoptosis and caspase‑3/GSDME‑dependent secondary pyroptosis, which was similar to the detection in our Lipin3 deficiency PTECs after cisplatin treatment. Our study also provided a novel molecule, Lipin3, that can regulate the GSDME-mediated pyroptosis via activating the p21. We have conducted a relatively in-depth study on the role of Lipin3 in regulating pyroptosis in cisplatin-induced AKI. However, cisplatin-induced AKI is a complex pathological process that involves multiple modes of cell death. Although our study has focused on pyroptosis, the potential role of other forms of cell death, such as ferroptosis, remains to be explored. This will be a key focus of our future research.

In summary, our study highlights the critical role of Lipin3 in protecting kidneys against AKI. The Lipin3 deficiency can further induce the mitochondrial dysfunction, apoptosis and Caspase 3/GSDME‑dependent secondary pyroptosis via Sirt1-p21-Caspase 3-GSDME pathway in PTECs and aggravated cisplatin induced AKI. Boosting Lipin3 expression could serve as a potential therapeutic approach for alleviating AKI progression.

## Supplementary Material

Supplementary figures and tables.
