# Supplementary material for: Lipin3 deficiency aggravates cisplatin induced acute kidney injury via activating Sirt1-p21-Caspase 3-GSDME pyroptosis pathway
Source: Int J Biol Sci. 2025 Aug 11;21(12):5185–205. doi: 10.7150/ijbs.110125 (PMC12435314; doi:10.7150/ijbs.110125)
Supplement: Supplementary file 1 — Supplementary figures and tables. [file ijbsv21p5185s1.pdf]

Lipin3 deficiency aggravates cisplatin induced acute kidney injury via activating Sirt1-p21-Caspase 3-GSDME pyroptosis pathway

Yu-Xing Liu[1,2,#], Hao Huang[1,2,3#], Fang Wang[4], Mei-Fang Zhao[2,3], Jie-Yuan Jin[2,3], Yi Dong[2], Qian Wang[2], Liang-Liang Fan[1,2,3*], Rong Xiang[1,2,3,4*]

1. Department of Nephrology, Xiangya Hospital, Central South University, Changsha, 410013, China;

2. Department of cell biology, School of Life Sciences, Central South University, Changsha, 410013, China;

3. Hunan Key Laboratory of Animal Models for Human Diseases, School of Life Sciences, Central South University, Changsha, 410013, China;

4. Department of Endocrinology, The Third Xiangya Hospital of Central South University, Changsha, Hunan 410013, China.

[#] These authors contributed equally to this work.

* Correspondence:

Liang-Liang Fan, Ph.D

Email: swfanliangliang@csu.edu.cn

Department of Cell Biology, The School of Life Sciences, Central South University, Changsha, 410013, China;

and

Rong Xiang, Ph.D.

Email: shirlesmile@csu.edu.cn

Department of Nephrology, Xiangya Hospital, Central South University, Changsha, 410013, China.

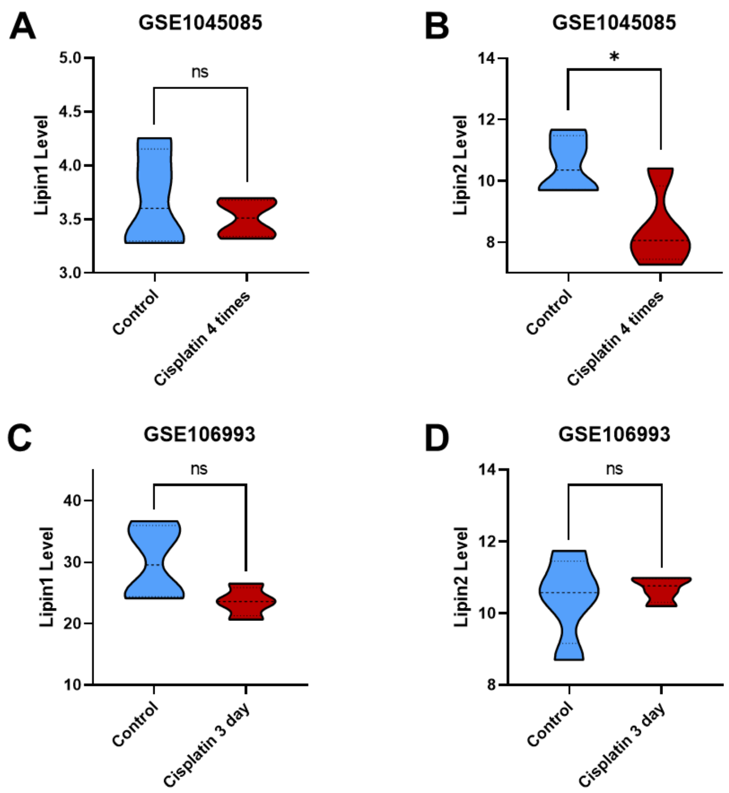

Supplementary Figure 1. The expression of *Lipin1* (A) and *Lipin2* (B) genes in human kidney organoids (GSE145085). The expression of *Lipin1* (C) and *Lipin2* (D) genes in mouse kidneys (GSE106993).

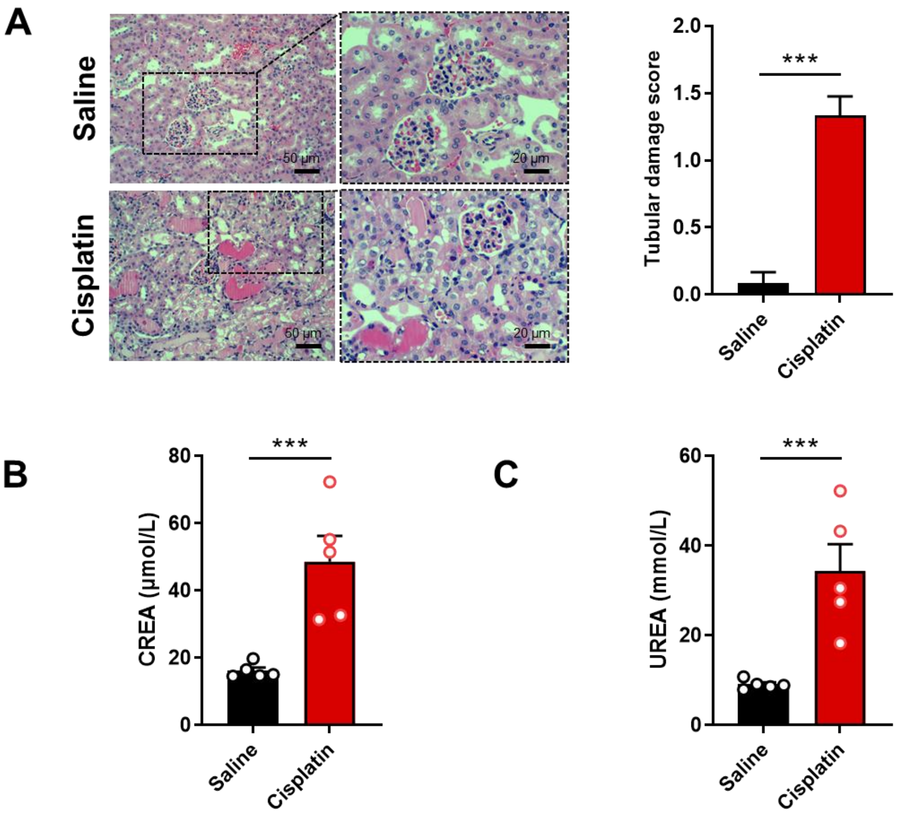

Supplementary Figure 2. (A) HE staining analysis showing the renal tubular injury in WT mice (n = 5) treated with or without cisplatin. Peripheral blood UREA (B) and creatinine (C) levels of WT mice (n = 5) treated with or without cisplatin.

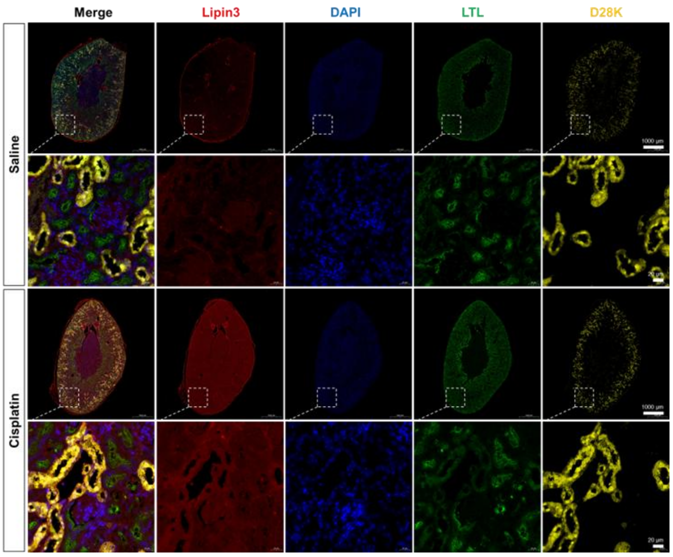

Supplementary Figure 3. Immunofluorescence shows the expression of Lipin3 in the mouse kidney. LTL, the proximal tubules marker; D28K, the distal tubules marker.

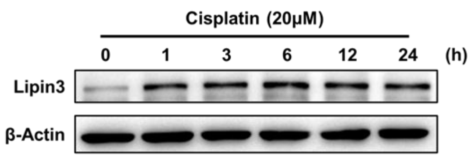

Supplementary Figure 4. WB analysis showing the expression levels of Lipin3 in HK2 cells treated with cisplatin at different time points.

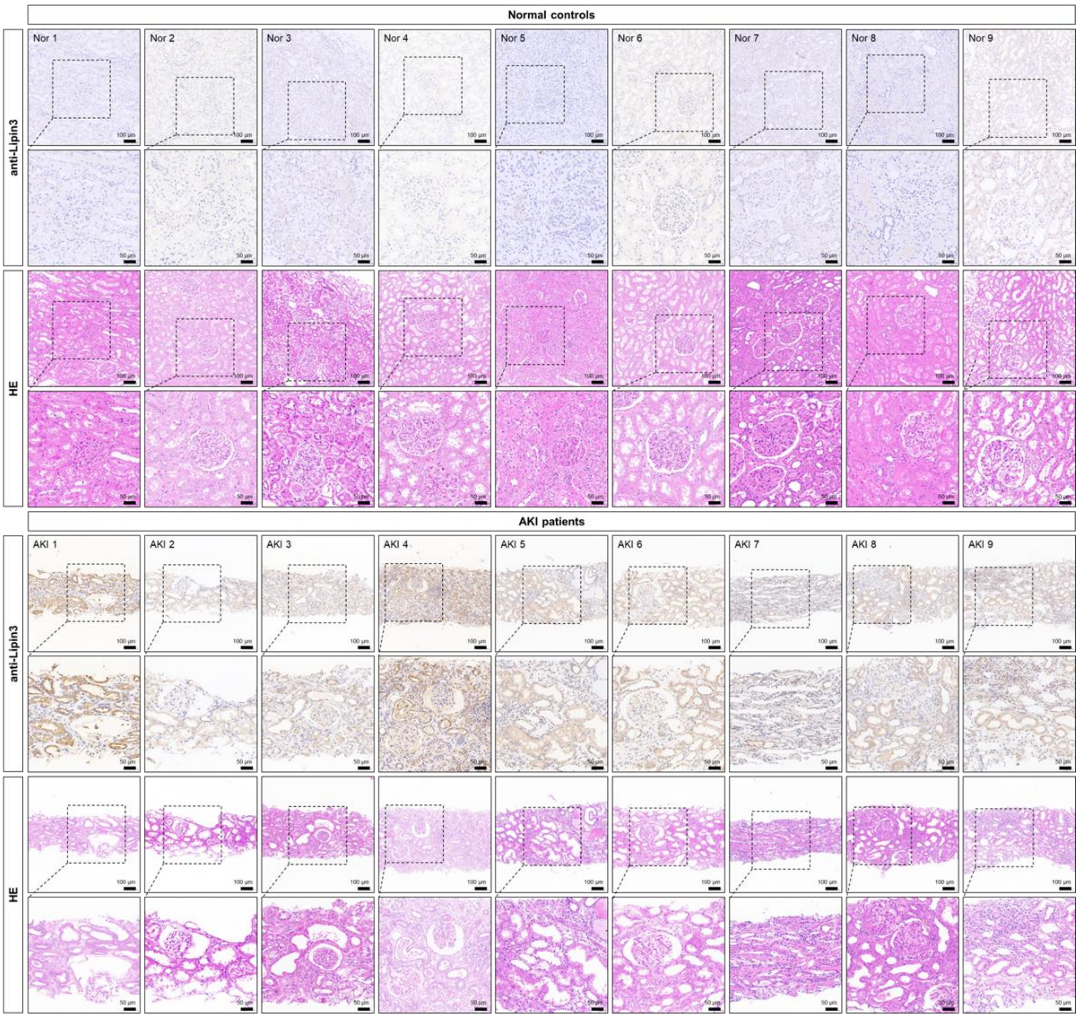

Supplementary Figure 5. IHC staining and HE staining showing the conditions of Lipin3 expression and renal tubular injury in kidney tissues from 9 healthy control subjects and 9 AKI patients.

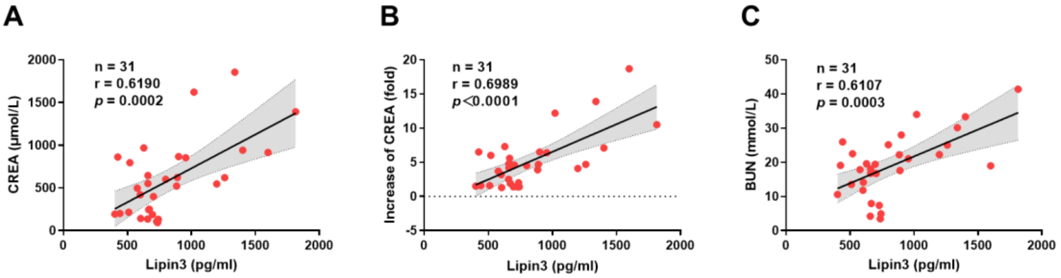

Supplementary Figure 6. Scatter plots showing the correlation between serum Lipin3 levels and serum creatinine levels (left), the fold increase in serum creatinine (middle) and serum BUN levels (right) in patients with nephrotoxic AKI. Correlation coefficient r and p value were calculated by the Spearman's rank correlation coefficient test.

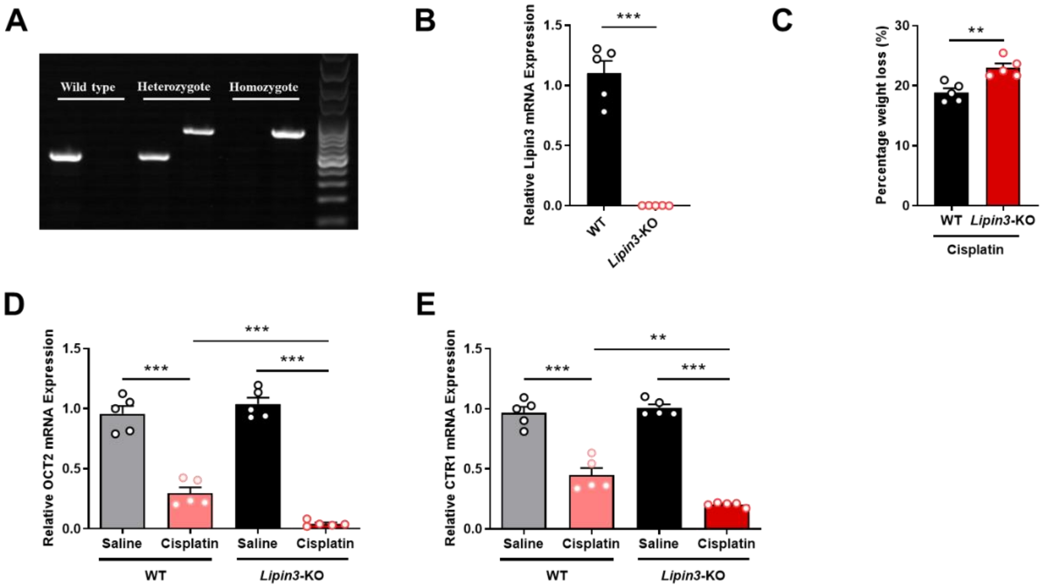

Supplementary Figure 7. The mouse genotypes were confirmed by agarose gel electrophoresis (A) and qPCR (B). (C) The results of body weight loss changes in WT and *Lipin3*-KO mice after cisplatin injection. mRNA levels of *OCT2* (D) and *CTR1* (E) in the kidney of mice in each group (n = 5).

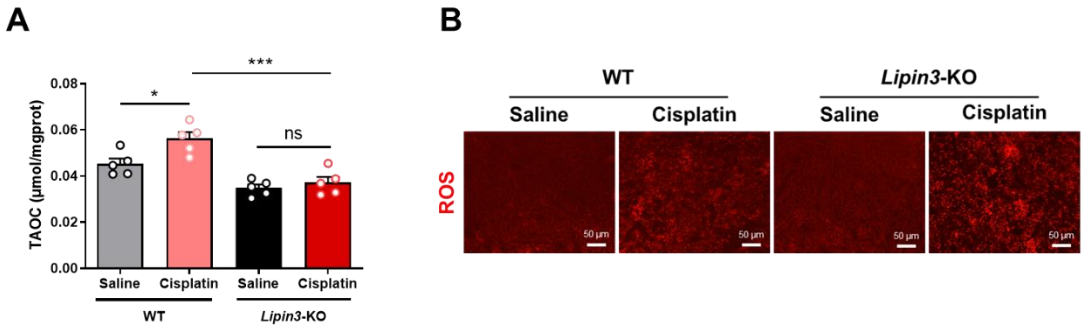

Supplementary Figure 8. (A) T-AOC levels in the in the kidney of WT mice (n = 5) and *Lipin3*-KO mice (n = 5) treated with or without cisplatin. (B) The DCF staining analysis showing the ROS levels in the kidney of WT mice (n = 5) and *Lipin3*-KO mice (n = 5) treated with or without cisplatin.

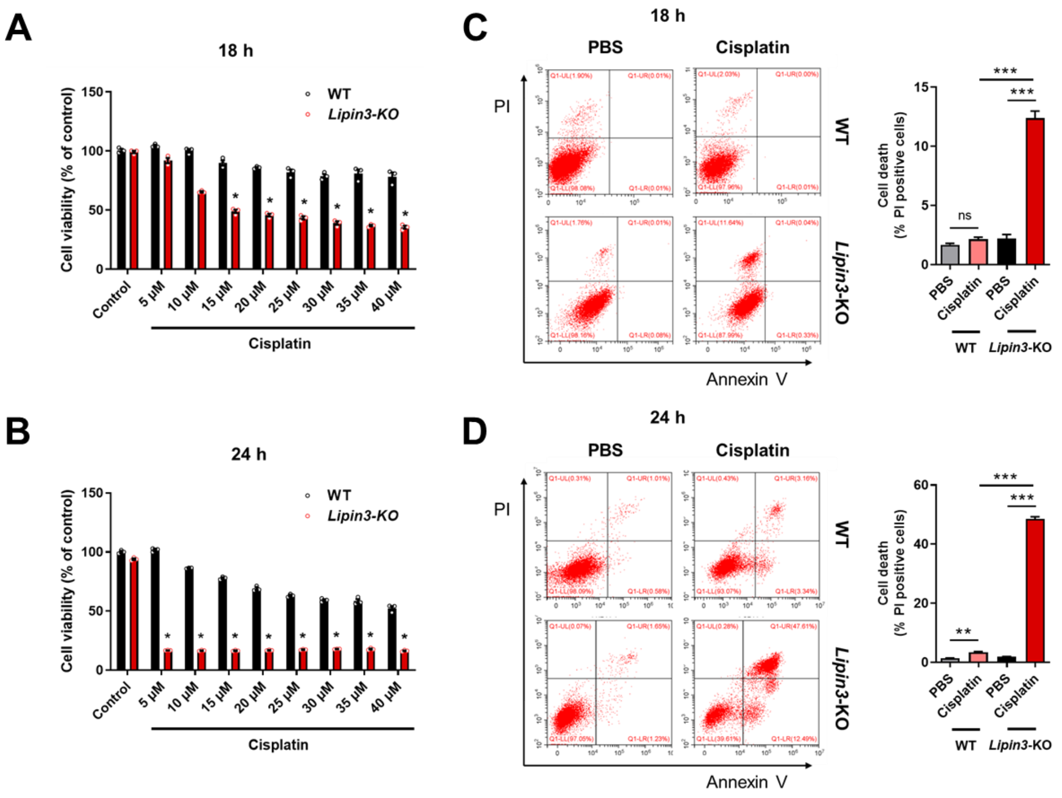

Supplementary Figure 9. CCK8 analysis showing the cell viability of WT and Lipin3-KO PTECs treated with different concentrations of cisplatin for 18h (A) and 24h (B), respectively. Flow cytometric analysis of PI- and Annexin V-FITC-stained PTECs treated with cisplatin for 18h (C) and 24h (D), respectively.

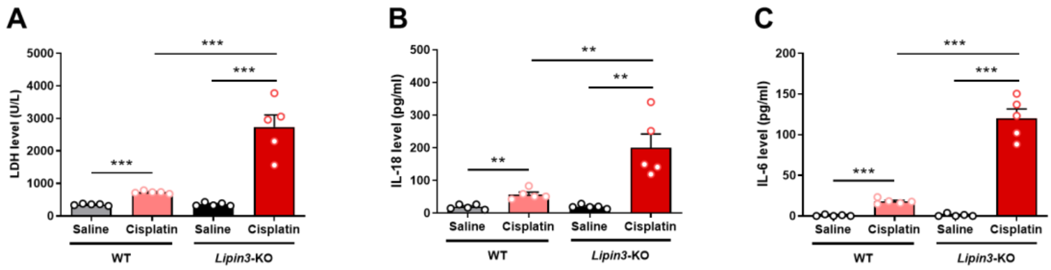

Supplementary Figure 10. LDH levels (A), IL-18 levels (B) and IL-6 levels (C) in the serum of WT mice and *Lipin3*-KO mice treated with or without cisplatin.

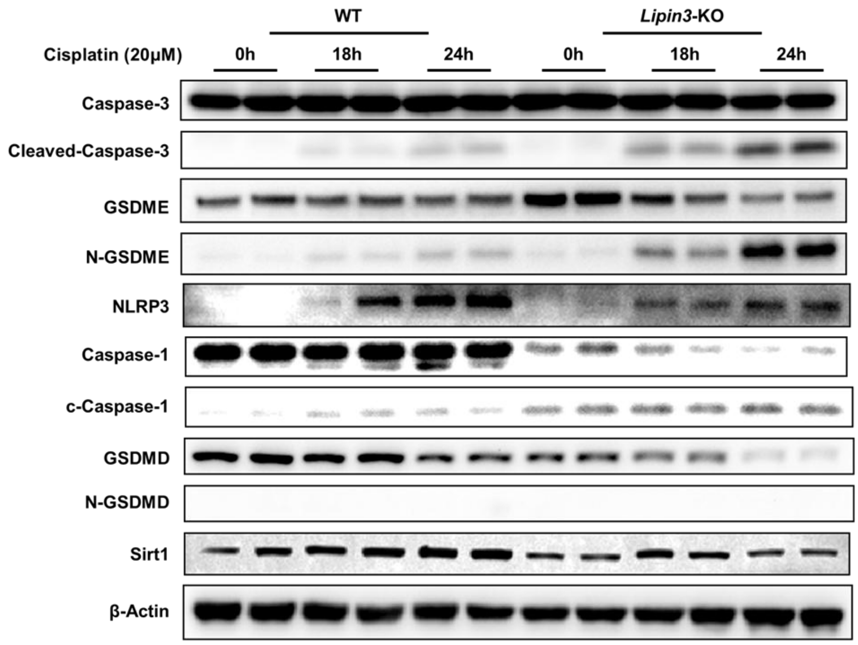

Supplementary Figure 11. WB analysis revealed the expressions of Caspase 3, cleaved-Caspase 3, GSDME, N-GSDME, NLRP3, Caspase 1, cleaved-Caspase 1, GSDMD, N-GSDMD, Sirt1 in WT and Lipin3-KO PTECs treated with cisplatin for different lengths of time.

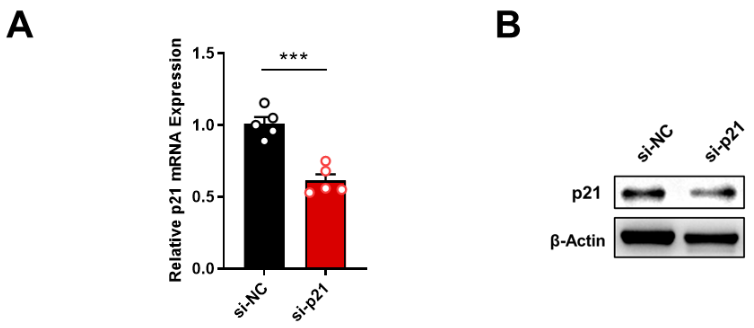

Supplementary Figure 12. The inhibitory efficiency of siRNA probes in *Lipin3*-KO primary cells was confirmed by qPCR (A) and WB analysis (B).

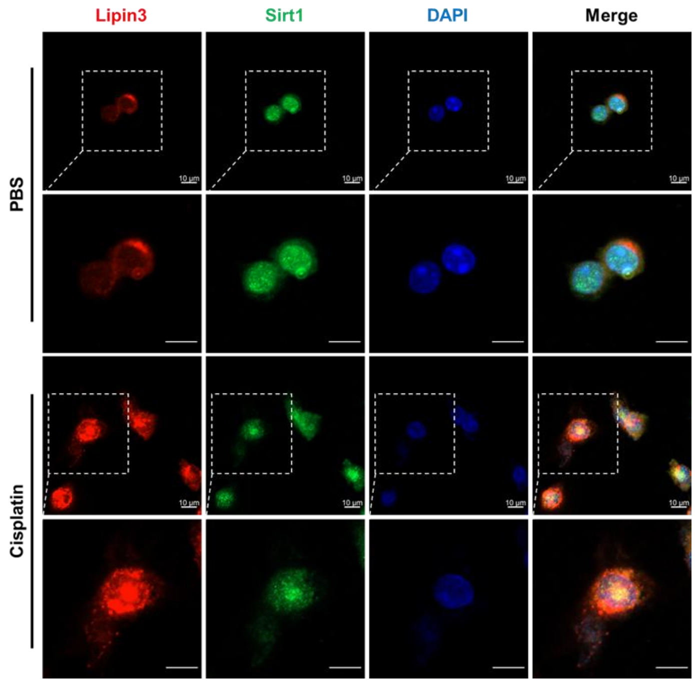

Supplementary Figure 13. Immunofluorescence staining showing the subcellular localization of Lipin3 and Sirt1 in HK2 cells with or without cisplatin treatment.

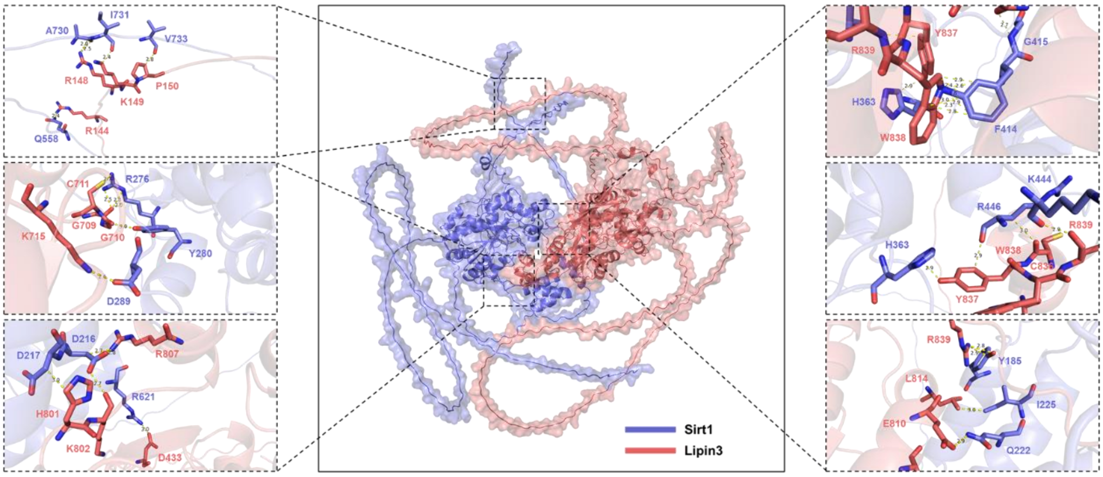

Supplementary Figure 14. Molecular docking studies reveal that Sirt1 is a potential interaction partner of Lipin3, with multiple binding sites identified between the two proteins.

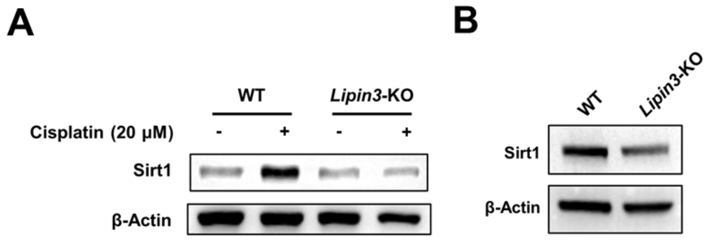

Supplementary Figure 15. (A) WB analysis revealed the expression of Sirt1 in WT and *Lipin3*-KO PTECs treated with or without cisplatin. (B) WB analysis revealed the expression of Sirt1 in WT and *Lipin3*-KO PTECs.

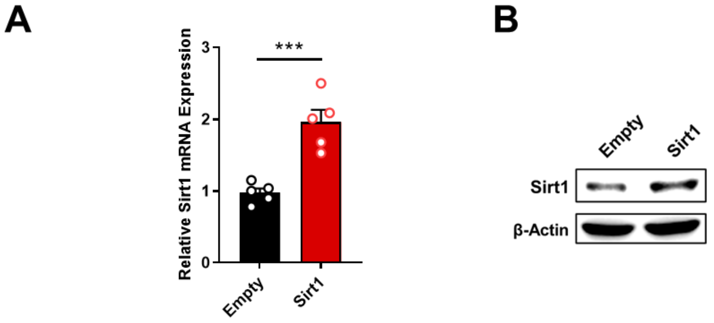

Supplementary Figure 16. The efficiency of plasmid-mediated Sirt1 up-regulation in *Lipin3*-KO primary cells was confirmed by qPCR (A) and WB analysis (B).

Table S1. Primers used in this study

Gene	Forward primer	Reverse primer	Species
Lipin3	TGGACTCCAGTTCTGAAGAGT	GTATGTCCTTAGGCTGCGATG	Mouse
ND1	CTAGCCTATCAGTTTACTCCATTCT	ATATGAAATTGTTTGGGCTACGG	Mouse
COX3	CCATCCTCCAAGCTTCAGAATAC	TGTCGTAGTAGGCAAACAATAAGG	Mouse
CYTB	CCTCCTATCAGCCATCCCATA	AAGCGAAGAATCGGGTCAAG	Mouse
CTR1	CCGGTTTGGTAATCAATACACCT	CCTCTCGGGCTATCTTGAGT	Mouse
OCT2	CTGGATGTTGGTGTTTCGATTT	AATCCCTACTGTTCTGCGATAG	Mouse
CAT	CTCCGGAACAACAGCCTTCT	ATAGAATGCCCGCACCTGAG	Mouse
GPX1	GCCACCGCGCTTATGAC	TCTCAAAGTTCCAGGCAACATC	Mouse
SOD2	GCTGGCTTGGCTTCAATAAG	GAATAAGGCCTGTTGTTCCTTG	Mouse
Sirt1	CAACAGCATCTTGCCTGATTTG	TAGGGCACCGAGGAACTAC	Mouse
p21	GAACATCTCAGGGCCGAAA	TCTCTTGCAGAAGACCAATCTG	Mouse

Table S2. Clinical characteristics of 54 patients.

Patient	Scr (μmol/L)	Scr increase from baseline (μmol/L)	Scr increase multiple (fold)	BUN (mmol/L)	UA (μmol/L)	AKI etiology
1	251	118	1.9	16.97	430.9	Nephrotoxins
2	1855	1722	13.9	30.14	946.8	Nephrotoxins
3	420	287	3.2	11.83	583.8	Nephrotoxins
4	285	152	2.1	22.24	384.9	Others
5	1392	1259	10.5	41.40	438.6	Nephrotoxins
6	399	312	4.6	16.74	474.0	Nephrotoxins
7	621	488	4.7	25.08	665.5	Nephrotoxins
8	497	364	3.7	17.84	345.5	Nephrotoxins
9	523	390	3.9	22.22	494.2	Nephrotoxins
10	862	729	6.5	19.11	653.7	Nephrotoxins
11	187	54	1.4	19.43	516.1	Nephrotoxins
12	216	83	1.6	13.50	217.7	Nephrotoxins
13	1622	1489	12.2	34.00	658.4	Nephrotoxins
14	645	512	4.8	16.76	461.8	Nephrotoxins
15	333	200	2.5	20.38	325.6	Sepsis
16	194	61	1.5	10.61	251.3	Nephrotoxins
17	795	662	6.0	22.53	645.7	Nephrotoxins
18	623	490	4.7	17.60	305.2	Nephrotoxins
19	854	721	6.4	21.07	511.9	Nephrotoxins
20	867	734	6.5	28.03	561.1	Nephrotoxins
21	552	419	4.2	18.08	461.1	Nephrotoxins
22	600	467	4.5	25.13	484.2	Nephrotoxins
23	942	809	7.1	33.37	324.7	Nephrotoxins

24	158	66	1.7	12.32	528.0	Sepsis
25	969	836	7.3	19.62	463.2	Nephrotoxins
26	916	867	18.7	18.99	439.8	Nephrotoxins
27	982	849	7.4	16.45	508.0	Others
28	306	173	2.3	15.05	617.0	Sepsis
29	574	441	4.3	18.28	492.0	Others
30	872	739	6.6	30.16	382.0	Others
31	267	174	2.9	17.19	657.0	Others
32	292	181	2.6	22.68	370.0	Others
33	82	16	1.2	7.63	432.0	Others
34	409	276	3.1	14.55	524.0	Others
35	617	484	4.6	14.24	585.0	Others
36	125	25	1.3	9.37	260.0	Others
37	839	706	6.3	18.91	623.0	Others
38	96	59	2.6	17.02	694.0	Others
39	237	104	1.8	10.85	257.7	Others
40	200	78	1.6	25.96	877.9	Nephrotoxins
41	197	64	1.5	20.27	611.5	Others
42	142	31	1.3	14.16	484.4	Nephrotoxins
43	108	28	1.4	7.39	194.1	Nephrotoxins
44	135	82	2.5	4.23	364.0	Nephrotoxins
45	113	29	1.3	5.92	195.6	Others
46	116	30	1.3	5.15	314.8	Others
47	121	27	1.3	5.10	409.4	Sepsis
48	174	41	1.3	7.84	402.5	Others
49	220	87	1.7	9.22	517.7	Sepsis

50	130	54	1.7	5.33	226.3	Others
51	94	46	2.0	3.48	842.0	Nephrotoxins
52	133	39	1.4	4.94	515.0	Nephrotoxins
53	548	415	4.1	22.29	519.0	Nephrotoxins
54	245	201	5.6	7.93	508.0	Nephrotoxins

BUN, blood urea nitrogen; Scr, serum creatinine; UA, uric acid.

Table S3. Clinical information of patients from 9 renal biopsy samples.

Patient	Age	Sex	Scr (μmol/L)	Pathological diagnosis	Coexistence of CKD
1	48	F	604.3	Acute tubulointerstitial nephritis	Unknown
2	32	M	381.9	Acute tubulointerstitial nephritis	Unknown
3	54	M	302.0	Acute glomerulonephritis	Unknown
4	44	M	699.0	Acute tubulointerstitial nephritis	-
5	21	M	267.0	Acute tubulointerstitial nephritis	-
6	58	M	153.8	Acute tubulointerstitial nephritis	-
7	36	M	533.0	Acute tubulointerstitial nephritis	DKD
8	62	M	188.2	Acute interstitial nephritis	-
9	53	M	431.1	Acute interstitial nephritis	-

CKD, chronic kidney disease; DKD, diabetic kidney disease.
